# Transcriptome Profiling of Cu Stressed Petunia Petals Reveals Candidate Genes Involved in Fe and Cu Crosstalk

**DOI:** 10.3390/ijms222111604

**Published:** 2021-10-27

**Authors:** Jinglei Wu, Kai Li, Jian Li, Henk Schat, Yanbang Li

**Affiliations:** 1 School of Agriculture and Biology, Shanghai Jiao Tong University, Shanghai 200240, China; 119150910083@sjtu.edu.cn (J.W.); 120150910079@sjtu.edu.cn (K.L.); 2 Institute of Plant Protection, Shandong Academy of Agricultural Sciences, Jinan 250100, China; lijian910@163.com; 3 Laboratory of Genetics, Wageningen University and Research, Droevendaalsesteeg 1, 6708 PB Wageningen, The Netherlands; henkschat@yandex.com

**Keywords:** petunia, petal, excess Cu, RNA-seq, Fe deficiency response, photosynthesis

## Abstract

Copper (Cu) is an essential element for most living plants, but it is toxic for plants when present in excess. To better understand the response mechanism under excess Cu in plants, especially in flowers, transcriptome sequencing on petunia buds and opened flowers under excess Cu was performed. Interestingly, the transcript level of FIT-independent Fe deficiency response genes was significantly affected in Cu stressed petals, probably regulated by basic-helix-loop-helix 121 (bHLH121), while no difference was found in Fe content. Notably, the expression level of bHLH121 was significantly down-regulated in petals under excess Cu. In addition, the expression level of genes related to photosystem II (PSII), photosystem I (PSI), cytochrome *b*_6_/*f* complex, the light-harvesting chlorophyll II complex and electron carriers showed disordered expression profiles in petals under excess Cu, thus photosynthesis parameters, including the maximum PSII efficiency (F_V_/F_M_), nonphotochemical quenching (NPQ), quantum yield of the PSII (ΦPS(II)) and photochemical quenching coefficient (qP), were reduced in Cu stressed petals. Moreover, the chlorophyll a content was significantly reduced, while the chlorophyll b content was not affected, probably caused by the increased expression of *chlorophyllide a oxygenase* (*CAO*). Together, we provide new insight into excess Cu response and the Cu–Fe crosstalk in flowers.

## 1. Introduction

Copper (Cu) is an essential micronutrient for most living plants as a cofactor in metalloproteins involved in many biological processes, such as ethylene signaling, reactive oxygen species (ROS) response, photosynthesis and respiration [1]. About 30% of leaf Cu is found in chloroplasts [2,3]. The most abundant Cu-containing protein in high plants is plastocyanin (PC), which localizes in chloroplast thylakoid lumen and functions as the electron carrier transporting electrons from the cytochrome *b*_6_/*f* complex (Cyt *b*_6_/*f*) to photosystem I (PSI) [4,5]. Under Cu limitation, chlorophyll biosynthesis and photosynthesis are inhibited causing chlorosis of young leaves and reproductive organs [6,7]. As a redox metal, Cu also can be toxic to plants by generating reactive oxygen species (ROS) under excess conditions. Therefore, the content of Cu in plant cells must be tightly regulated. Several proteins have been reported involved in Cu detoxification, such as metallothioneins (MTs), heavy metal P-type ATPases (HMA5I and HMA5II) and Copper chaperons (CCH) [8,9,10]. However, the transcription factors (TFs) involved in Cu detoxification have not been identified yet. The Cu deficiency response in Arabidopsis roots is controlled by squamosa promoter binding protein-Like7 (SPL7), which is constitutively expressed in roots [11]. Under Cu deficiency, SPL7 activates expression of copper transporter2 (COPT2), ferric reductase oxidase 4 (FRO4) and FRO5 which promotes Cu uptake into the roots [11,12].

In addition to Cu, iron (Fe) is also an essential micronutrient for all living plants. Up to 80% of leaf Fe is found in chloroplasts [2]. Fe serves as a cofactor in metalloproteins that participate in photosynthesis, such as Psb proteins in PSII, Pet proteins in cyt *b*_6_/*f*, Psa proteins in PSI and ferredoxin (FD) [13]. Thus, Fe deficiency causes severe damage to photosynthesis. Fe homeostasis is tightly regulated by numerous basic-helix-loop-helix (bHLH) TFs, including the bHLH Ib subgroup TFs (bHLH38, bHLH39, bHLH100 and bHLH101), the bHLH IVc subgroup TFs (bHLH034, bHLH104, bHLH105/ILR3 and bHLH115), the bHLH IVb subgroup TFs (PYE, bHLH121 and bHLH11) and Fer-like iron deficiency-induced transcription factor (FIT). Under Fe deficiency, FIT interacts with bHLH Ib TFs to activate the transcription of Iron-regulated transporter1 (ITR1) and Ferric reductase oxidase2 (FRO2) to enhance the uptake of Fe into the roots [14,15]. FIT is especially expressed in roots, whereas the Fe homeostasis in shoots is controlled by FIT-independent genes, including *bHLH Ib TFs*, *Brutus* (*BTS*), *BTS-like*(*BTSL*), *PYE*, *FRO3*, *Oligopeptide transporter3* (*OPT3*), *Nicotianamine synthases4* (*NAS4*) and *Zinc-induced facilitator* (*ZIF1*) [16], and the expression of these genes is regulated by bHLH121 [17].

Crosstalk between Fe and Cu has been reported [18,19,20,21]. Under Fe deficiency, FIT and bHLH Ib TFs complex directly bind to the promoter of *COPT2*, *FRO4* and *FRO5* to activate their expression for the uptake of Cu to alleviate the Fe deficiency stress [18]. In addition, under Fe deficiency, Fe responsive genes, such as *Iron mans* (*IMAs*) and *OPT3*, were activated by SPL7 in Arabidopsis roots [21]. Besides this, genes involved in both Fe homeostasis and Cu homeostasis also have been found in some species by transcriptome sequencing [19,20,21]. However, most of these studies were focused on the response in roots under metal deficiency conditions, and little is yet known about the response mechanism in the above-ground tissues, especially under excess Cu condition. It has been reported that excess Cu has serious impact on the flower pigmentation, petal senescence, flowering period and flower yield [22,23,24,25]. However, the response mechanism in petals under excess Cu was largely unknown. Moreover, several Cu homeostasis related genes, such as *HMA5*, *COPT1*, *COPT6*, Natural resistance-associated macrophage proteins1 (*NRAMP1*) and *NRAMP3* show different expression patterns in petals and leaves, indicating that Cu response mechanism might be different in petals and leaves [26,27].

Here, we investigated the response genes in petunia petals under excess Cu by transcriptome sequencing. The main effects of excess Cu on petunia petals were Fe–S cluster assembling and photosynthesis-related, with most of the genes affected also being responsive to Fe deficiency. The expression level of petunia *bHLH121-like* was down-regulated in petals under excess Cu, and unaffected by Fe deficiency, suggesting that bHLH121-like protein might be involved in Cu homeostasis in petunia petals. 

## 2. Results

### 2.1. Dry Weight and Element Content in Petunia Petals under Excess Cu Treatment

**Figure 1:**
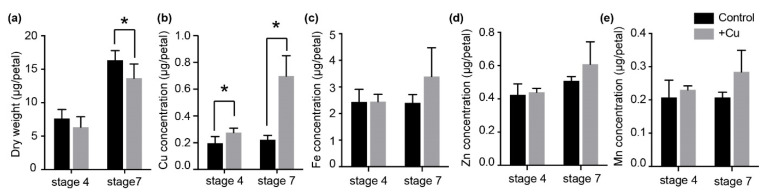
Dry weight and element concentrations in stage 4 and stage 7 petunia petals in plants that were unexposed (Control), or exposed to 40 μM Cu (+Cu). (**a**) Dry weight of stage 4 and stage 7 petal upon exposure to excess Cu. Bars are mean ± SD (*n* = 6). (**b**) Cu, (**c**) Fe, (**d**) Zn and (**e**) Mn content in petunia petals under excess Cu. Bars represent mean ± SD (*n* = 4 × 3 petals). * indicates significant differences by Tukey’s HSD test (*p* < 0.05).

Upon exposure to excess Cu, the dry weight (DW) of opened flowers (stage 7) was significantly lower than the control, while there was no significant difference between flower buds (stage 4) (Figure 1a). The Cu concentration in stage 4 and stage 7 petals was significantly increased under excess Cu treatment (Figure 1b), whereas the Fe, Zn and Mn concentrations were not significantly affected by the presence of 40 μM Cu (Figure 1c–e). 

### 2.2. Transcriptome Profiling Analysis

**Figure 2:**
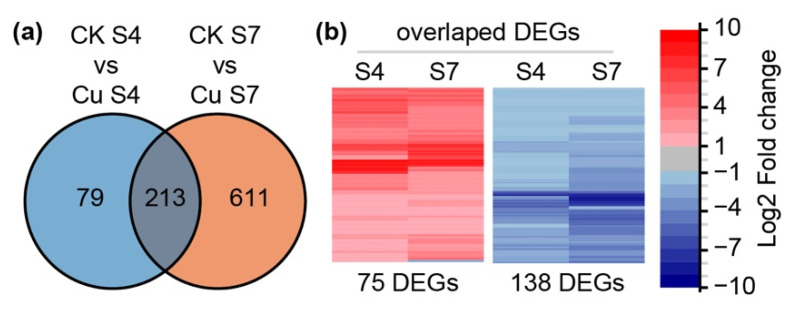
Differentially expressed genes (DEGs) in stage 4 and stage 7 petals after exposure to 40 μM Cu. (**a**) Venn diagram showing the number of DEGs, fold change > 2 and FDR < 0.05, in stage 4 and stage 7 petals. (**b**) Heatmap showing the log2-fold change of DEGs shared by stage 4 and stage 7 petals.

In order to study the regulatory mechanism in petunia petal under excess Cu, RNA-seq was performed using stage 4 and stage 7 petals from petunia treated with 40 μM Cu. The comparison of these transcriptomes identified 292 genes (106 up-regulated and 186 down-regulated) and 824 genes (303 up-regulated and 521 down-regulated) with significant differential expression in Cu treated stage 4 petals and stage 7 petals, respectively, using fold-change > 2 and false discovery rate correction adjusted (FDR) <0.05 as a cutoff (Figure 2a). Among those differentially expressed genes (DEGs), 213 genes were found in both stage 4 and stage 7 transcriptomes. Except for one gene, encoding a Glycine-rich protein, all of them had the same expression profile under excess Cu treatment, of which 74 were up-regulated and 138 were down-regulated (Figure 2b).

To verify the RNAseq results, petunia genes were selected from stage 4 and stage 7 transcriptomes for real-time RT-PCR (qRT-PCR) analysis. The expression patterns of these selected genes were in line with the RNA-seq results, and there was a strong positive correlation between RNAseq and qRT-PCR results in both stage 4 and stage 7 (Figures S1 and S2), suggesting that the RNA-seq data were reliable.

### 2.3. KEGG and GO Enrichment Analysis

**Figure 3:**
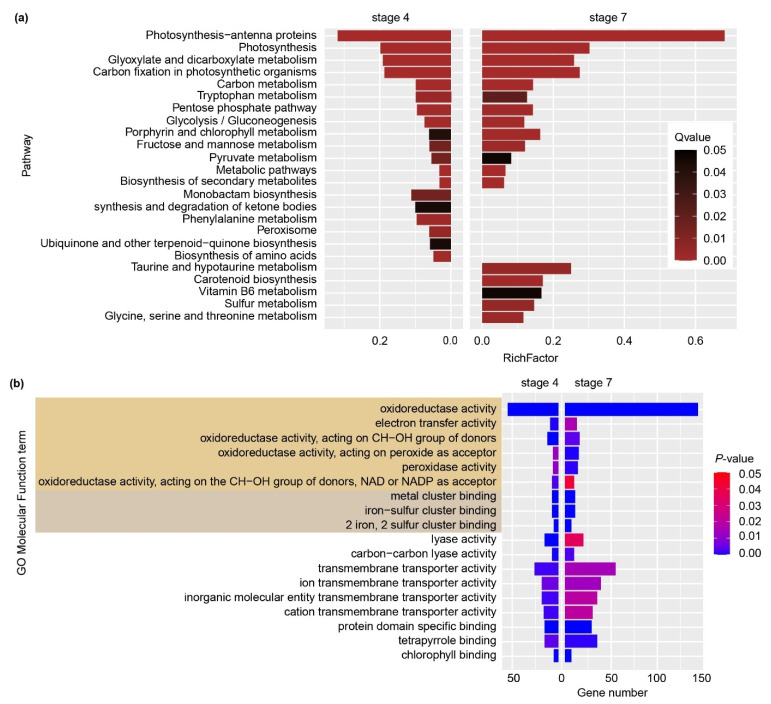
KEGG and Gene ontology (GO) enrichment analysis of transcripts significantly differently expressed in stage 4 and stage 7 petunia petal after exposure to excess Cu. (**a**) Enriched KEGG pathway in petunia petal upon excess Cu exposure by using *p*-value < 0.05 as a cut-off. (**b**) Enriched GO terms in molecular function ontology on level 3 to level 6 shared by stage 4 and stage 7 petal transcriptomes. Terms related to “oxidoreductase activity” and “metal cluster binding” are marked with colored backgrounds.

To gain more information about the response mechanism in petunia petals under excess Cu treatment, Kyoto Encyclopedia of Genes and Genomes (KEGG) pathway enrichment analysis was performed. “Photosynthesis-antenna proteins” and “Photosynthesis” were the top2 enriched KEGG pathways in both stage 4 and stage 7 petals. Beside these two pathways, “carbon fixation in photosynthetic organisms”, “carbon metabolism”, “porphyrin and chlorophyll metabolism”, all of them being photosynthesis-related, were all shared by stage 4 and stage 7 petals (Figure 3a).

Gene ontology (GO) annotation enrichment was also performed with *p*-value < 0.05 as a cut-off (Figure 3b). Both stage 4 and stage 7 petals appeared to be enriched in “oxidoreductase activity”, “electron transfer activity”, “metal cluster binding”, “transmembrane transporter activity”, “chlorophyll binding” and “lyase activity”, within in the molecular function category. Notably, most of these categories are closely related to photosynthesis and heavy metal homeostasis.

### 2.4. Expression Pattern of Cu Homeostasis Genes

**Figure 4:**
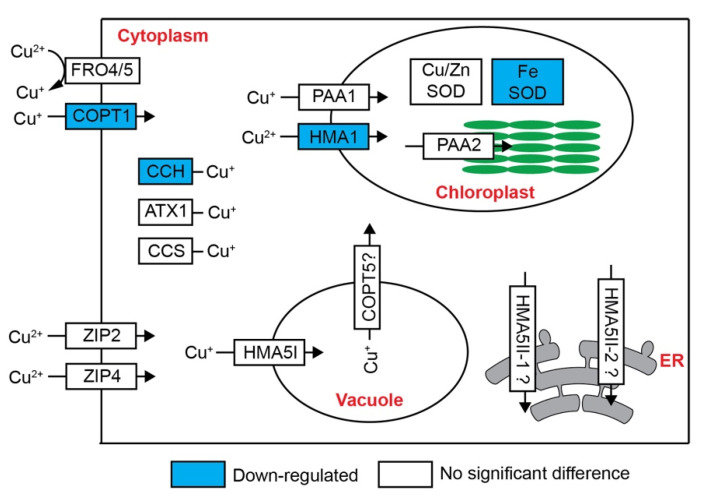
Expression pattern of genes relate to Cu transport and distribution in petunia petal under excess Cu. Blue box indicates down-regulated genes, whiter box indicates no significant difference after excess Cu treatment. FRO, ferric reductase oxidase; COPT, copper transporter; ZIP, zinc and iron regulated transporter proteins; HMA, heavy metal P-ATPase; CCH, copper chaperone; CCS, copper chaperone for superoxide dismutase; ATX1, antioxidant 1; PAA1, HMA6; PAA2, HMA8; ER, endoplasmic reticulum.

Cu detoxication genes in roots and shoots have been studied in Arabidopsis, rice and *Silene vulgaris* [9,10,28]. We investigated the expression of known Cu homeostasis genes in petunia petal under excess Cu treatment. Surprisingly, only *COPT1*, encoding COPT-family transporter, *CCH*, one Cu chaperone, and *HMA1*, encoding chloroplast copper transporter, were differently expressed in petunia under excess Cu (Figure 4, Table 1).

### 2.5. Expression Profile of Fe Homeostasis Genes

**Figure 5:**
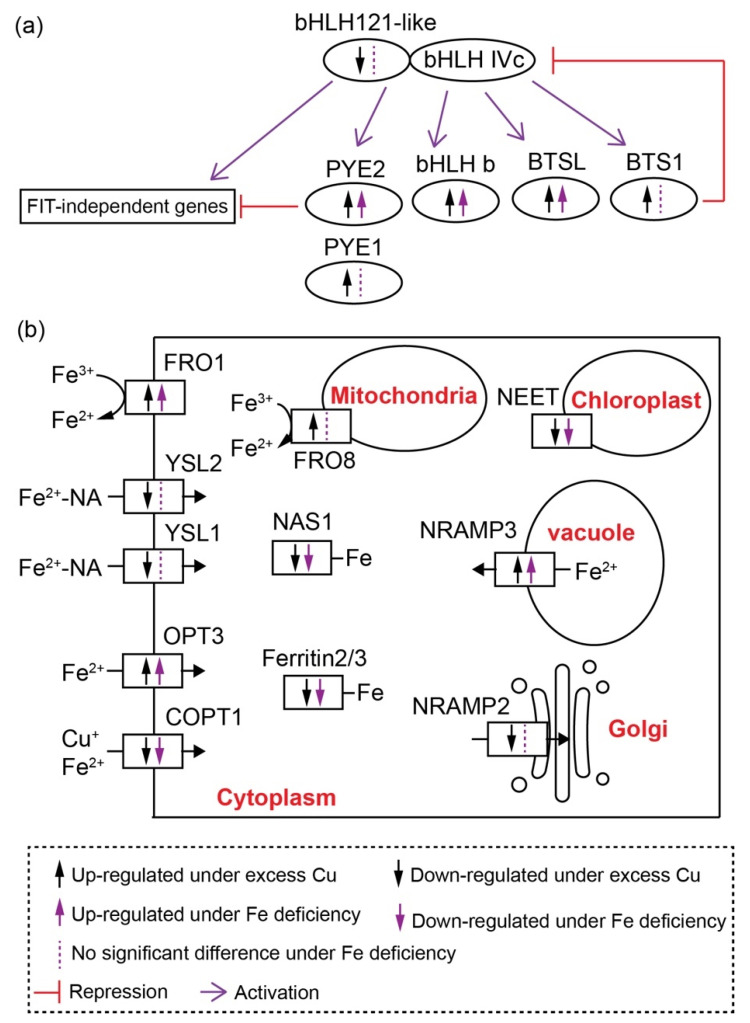
Schematic diagram of genes involved in Cu–Fe crosstalk in petunia petal: (**a**) expression profile of transcription factors involved in Fe homeostasis, (**b**) expression pattern of genes involved in Fe transport and distribution in petunia petals. bHLH, basic-helix-loop-helix; PYE, popeye; BTS, Brutus; BTSL, Brutus-like; NRAMP, Natural resistance-associated macrophage proteins; NAS, Nicotianamine synthases; FRO, ferric reductase oxidase; YSL, Yellow stripe-like proteins; OPT3, Oligopeptide transporter3; COPT1, copper transporter1.

In contrast to Cu homeostasis related genes, many more genes involved in Fe deficiency response were differently expressed in petunia petal transcriptomes (Table 1, Figure 5).

Unlike that of Arabidopsis, the *Petunia axillaris* genome encodes four proteins belonging to the bHLH IVb subfamily, including two Pypeye (PYE) orthologous proteins and two proteins closed related to bHLH121 (Figure S3). The expression levels of these two *PYEs*, named *PYE1* and *PYE2*, were both elevated under excess Cu in petunia petals (Table 1). In contrast with these *PYEs*, the ortholog of *bHLH121* was repressed under excess Cu treatment in both stage 4 and stage 7 petals (Table 1). Two *bHLH38/39/100/101* orthologs were found in the DEG lists, and both were more highly expressed in petals after excess Cu treatment (Table 1, Figure S4).

Members of the Brutus (BTS) hemerythrin E3 ligase family, e.g., Brutus-like1 (BTL1) and BTL2 in *Arabidopsis thaliana*, also play an important role in the Fe deficiency response [29,30]. Unlike in Arabidopsis, three BTS and one BTL homologs were identified in the *Petunia axillaris* genome (Figure S5). Two, one *BTS* and one *BTL*, were overexpressed in stage 4 and stage 7 petunia petals under Cu treatment (Table 1, Figure 5).

Two myeloblastosis (MYB) subgroup 3 proteins, MYB10 and MYB72, are involved in Fe homeostasis through the driving the expression of *nicotinamine synthase 4* (*NAS4*) [31,32]. Only one MYB protein belonging to MYB subgroup 3, including MYB10, MYB72, MYB63 and MYB58, was found in the *Petunia axillaris* genome, and it was up-regulated in Cu-treated stage 7 petals (Table 1 and Figure S6).

The ferrous transporter IRT1 and ferric chelate reductase2 (FRO2) are two major Fe acquisition genes, which are strongly upregulated under Fe deficiency in Arabidopsis [33,34,35]. *Petunia axillaris* encodes three AtIRT orthologous proteins, all of them not expressed in flowers (Table 1 and Figure S7). Three genes encoding FRO proteins were found in stage 4 and stage 7 transcriptomes, two belonging to the AtFRO1/2/3 subgroup and one to the FRO8 subgroup (Figure S8). The expression of all of these three *FROs* was induced by excess Cu in petals (Table 1). One oligopeptide transporter OPT3 is also involved in Fe transport [36]. Petunia *OPT3* was more expressed upon exposure to Cu excess in petals (Table 1, Figure 5 and Figure S9). Yellow stripe-like proteins (YSLs) mediate metal-nicotinamine (NA) transport [37,38,39,40]. Two *YSL* genes, all encoding proteins belonging to YSL1/2/3 subfamily, were up-regulated in petals under excess Cu treatment (Table 1, Figure 5 and Figure S10). The *Petunia axillaris* genome encodes two NAS proteins, one of them showing lower expression under excess Cu treatment, while the other was barely expressed, in S4 and S7 petals (Table 1, Figure 5).

Two genes encoding Natural resistance-associated macrophage proteins (NRAMPs) were found in our transcriptomes. According to the phylogenetic analysis, they were related to NRAMP2/5 and NRAMP3/4 in Arabidopsis, respectively. NRAMP2, belonging to AtNRAMP2/5 subgroup, only showed lower expression in stage 7 petunia petals under Cu stress, while no difference was found in stage 4 petals. NRAMP3 belonging to AtNRAMP3/4 subgroup showed enhanced expression in petunia petals under Cu stress. (Table 1, Figure 5 and Figure S11).

Ferritins are the major proteins involved in Fe storage. The *Petunia axillaris* genome encodes three Ferritin (Fer) proteins; notably, the expression of all of these three genes was decreased in stage 4 and stage 7 upon exposure to Cu excess (Table 1, Figure 5 and Figure S12).

Together, all the results indicated that the network controlling the Fe deficiency response was significantly affected in petunia petals under excess Cu.

### 2.6. Excess Cu Affects Photosynthesis and Fe-Dependent Proteins in Petal

**Figure 6:**
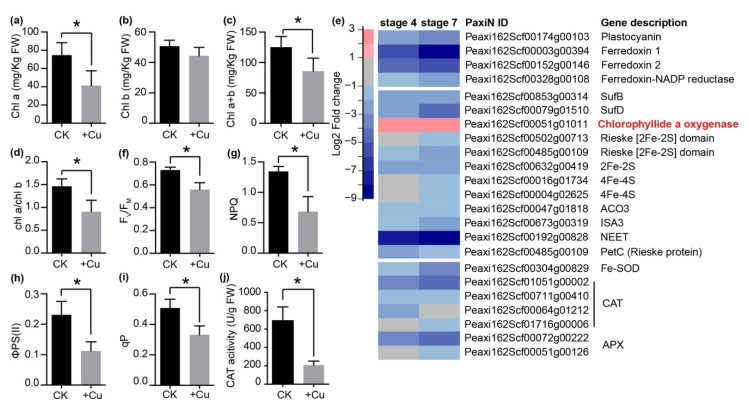
Effects of excess Cu treatment on photosynthesis in petunia petals. (**a**–**d**) Content of chlorophyll a (**a**), chlorophyll b (**b**), chlorophyll a+b (**c**) and chl a/b ratio (**d**) in stage 4 petunia petals. Bars are means ± SD (*n* = 5 petals). (**e**) Expression profiles of Fe–S metabolism related genes under the GO:0051536 item, which were enriched in stage 4 and stage 7 petals. (**f**) F_V_/F_M_, maximum potential PS II efficiency; (**g**) NPQ, non-photochemical quenching value; (**h**) ΦPS(II), actual PS II efficiency; and (**i**) qP, photochemical quenching value was measured in stage 4 petals under excess Cu. Bars are means ± SD (*n* = 6 petals). (**j**) CAT activity in petunia stage 4 petals upon Cu excess. Bars are mean ± SD (*n* = 3). * indicates significant differences (*p* < 0.05) using Tukey’s HSD test.

The KEGG and GO enrichment results led us to investigate the effects of excess Cu on photosynthesis-related symptoms. The chlorophyll a content, chlorophyll a+b content and chl a/b ratio was significantly decreased, while the chlorophyll b content was not affected by excess Cu in petals (Figure 6a–d). Accordingly, the expression level of *HEMA2*, encoding glutamyl-tRNA reductase, was down-regulated in petals under excess Cu, while *chlorophyllide a oxygenase* (*CAO*), encoding enzyme for chlorophyll b biosynthesis, was induced (Figure 6e and Table S1).

In addition, the F_V_/F_M_, nonphotochemical quenching (NPQ), ΦPS(II) and qP values were significantly decreased in the petals under excess Cu (Figure 6f–i). All these results agreed with the RNA-seq result that genes involved in electron transport, including *plastocyanin* (*PC*), ferredoxin-NADP reductase (*FNR*) and two ferredoxin (*FD*) genes, were significantly down-regulated in petals under excess Cu (Figure 6e). Moreover, the photosynthesis changes also were in agreement with the KEGG pathway enrichment results that the expression of genes related with photosystem II (such as *PsbO*, *PsbP*, *PsbQ*, *PsbW* and *PsbY*), photosystem I (such as *PsaD*, *PsaF*, *PsaH*, *PsaK*, *PsaI* and *PsaN*), the cytochrome *b*_6_/*f* complex (*PetC*) and the light-harvesting chlorophyll II complex were also markedly down-regulated by excess Cu treatment in petunia petals (Figure S13).

Iron–sulfur (Fe–S) clusters are the most abundant iron cofactors in the plant, and the Fe–S cluster biosynthesis and mobilization inside the cell is tightly controlled, to avoid toxicity of free or loosely bound iron [41]. According to the GO enrichment analysis, Fe–S cluster binding is significantly enriched in stage 4 and stage 7 petals (Figure 3b). The expression profile of genes involved in Fe–S cluster metabolism was investigated in more detail (Figure 6e). Except for *CAO*, all the genes involved in Fe–S cluster binding were down-regulated in Cu-stressed petals, including those encoding two subunits of the Fe–S scaffold complex (*SurB* and *SurD*) and *NEET* (Figure 6e).

Fe metabolism is tightly linked with ROS homeostasis in plants, especially with regard to Fe-containing proteins, such as catalase (CAT), ascorbate peroxidase (APX) and Fe superoxide dismutase (FeSOD). *CAT*, *APX* and *FeSOD* were all down-regulated in petunia petals under Cu excess (Figure 6e). Notably, the *Petunia axillaris* genome encodes four CAT proteins and all the four *CAT* genes were significant down-regulated in petunia petals under excess Cu (Figure 6e). Accordingly, the CAT activity was significantly lower in petals under excess Cu (Figure 6j).

### 2.7. Expression Levels of DEGs in Petunia Petals under Fe-Deficiency Treatment

**Figure 7:**
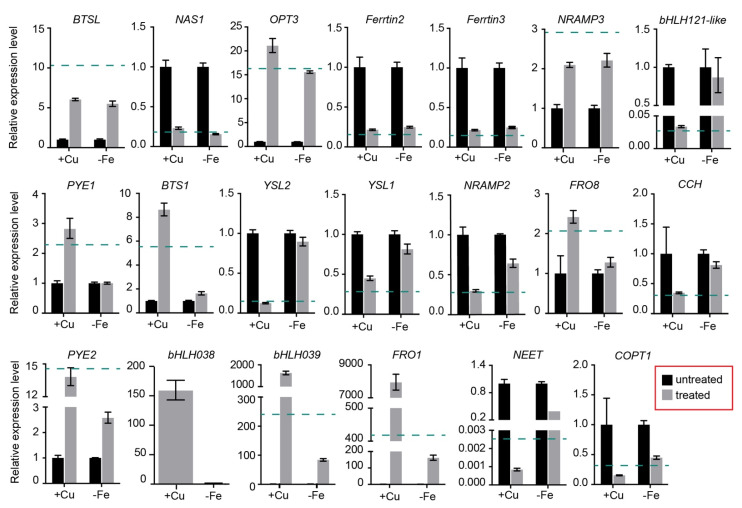
The relative expression level of genes involved in Fe and Cu homeostasis in petunia petals under Fe deficiency or excess Cu. Bars indicate the relative expression level determined by qRT-PCR in petunia petals exposed to 40 μM Cu (+Cu) or 0 μM Fe (-Fe). EF1α as the reference gene. Values (mean ± SD, *n* = 3) are normalized to the gene expression level in untreated petals (set as 1). Since bHLH038 was barely expressed in untreated petals (Cq value > 35), we used the relative expression value of -Fe as 1. The green dash line indicates the fold change value of genes in petunia stage 7 petal transcriptome.

To gain more information about the underlying mechanisms in petunia petal response to Cu stress, the expression pattern of Fe homeostasis related genes in petunia petals under Fe deficiency was studied using real-time PCR (qRT-PCR). Petunias were grown in -Fe half Hoagland’s nutrient solution and the total RNA was extracted from stage 7 petals for the qRT-PCR analysis (Figure 7).

The expression level of *BTSL*, *NAS1*, *OPT3*, *FER2*, *FER3* and *NRAMP3* was affected by Fe deficiency (fold change > 2), and the fold change was not different from that in excess Cu treated petals. The expression level of *bHLH121-like*, *PYE1*, *BTS1*, *YSL1*, *YSL2*, *NRAMP2*, *FRO8* and *CCH* was not significantly affected by Fe deficiency treatment (fold change < 2), indicating these genes are responsive to excess Cu, rather than Fe deficiency. The expression level of *COPT1*, *PYE2*, *bHLH038*, *bHLH039*, *FRO1* and *NEET* was differently expressed in both Fe deficiency treated and excess Cu treated petals, while the fold change in petals under excess Cu treatment was higher than that under Fe deficiency, indicating these genes might be involved in both Fe deficiency and Cu stress response pathways (Figure 7).

**Figure 8:**
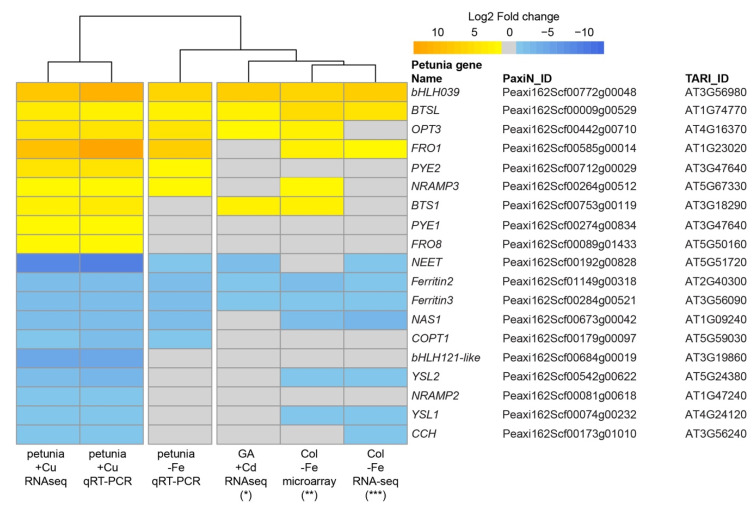
Heatmap showing the expression profile of genes related to Fe homeostasis in petunia petals under Cu excess, petunia petals under Fe depletion, *N. caerulescens* shoots under Cd excess and Arabidopsis shoots under Fe deficiency. The value of petunia is log2 fold change of qRT-PCR. The log2 fold change of genes with fold change < 2 or FDR (*p*-value for Col) >0.05, all been marked as 0. * data was download from Halimaa et al. [42]. ** data was download from Schuler et al. [44]. *** data was download from Kastoori Ramamurthy et al. [21].

Heavy metal-induced Fe deficiency responses in above-ground tissues have also been observed in cadmium-stressed *Noccaea caerulescens* shoots [42,43]. The expression profiles of Fe homeostasis-related genes in both transcriptomes were also compared (Figure 8). The transcript levels of *bHLH039*, *BTSL*, *OPT3*, *NEET*, *FER2* and *FER3* were significantly affected by excess Cd treatment in *N. caerulescens* shoots and Fe deficiency treatment in Arabidopsis shoots (fold change > 2 and FDR < 0.05). The transcript levels of *bHLH121-like*, *PYE1*, *FRO8* and *NRAMP2* were not affected by excess Cd treatment in *N. caerulescens* shoots and by Fe deficiency treatment in Arabidopsis shoots, confirming that these genes may specifically respond to Cu stress in petunia petals. On the other hand, the transcript fold-change of *NEET* in petunia petals under Fe deficiency is similar to that in excess Cd-treated *N. caerulescens* shoots and Fe deficiency-treated Arabidopsis shoots, and markedly lower than that in excess Cu-treated petunia petals.

## 3. Discussion

In this study, we investigated the transcriptional response to excess Cu in petunia petals, and provided new insights into the crosstalk between iron and copper homeostasis. Interestingly, the primary effects of excess Cu in petunia petals are related to Fe deficiency, such as disordered expression profiles of Fe deficiency response genes, the blocking of Fe-S cluster metabolism, and a dysfunctioning photosynthesis.

### 3.1. Fe Deficiency Response in Petunia Petals under Excess Cu

Two sets of Fe-regulated genes have been described in Arabidopsis, i.e., FIT-dependent and FIT-independent [16]. The FIT-dependent pathway is controlled by the FIT and bHLH Ib subfamilies of TFs, including bHLH38/39/100/101. FIT interacts with bHLH Ib TFs to activate the expression of *IRT1* and *FRO2*, which are involved in Fe uptake into the root [45]. Certainly, this is not the case in petunia petals, because the orthologs of *FIT* and *IRT* are not expressed in petals (Table 1). Thus, the Fe deficiency response in petunia petals is FIT-independent pathway, like in Arabidopsis shoots [16]. Among the known Fe deficiency response genes, the expression levels of *bHLH39*, *BTSL*, *OPT3*, *FRO1*, *NRAMP3*, *NEET*, *FER2*, *FER3* and *NAS1* were significant affected by Fe deficiency or excess Cu in petals. Interestingly, except for *PYE2*, all the other known Fe-responsive genes that responded to Cu stress in petunia petals, were also significantly affected in Cd-stressed *N. caerulescens* shoots and/or Arabidopsis shoots upon Fe deficiency, indicating that the Fe deficiency response in the shoot, triggered either directly by Fe deficiency, or by Cd or Cu excess, is conserved among different species.

*FER2* and *FER3* encode ferritin proteins, which are an iron source for maintaining the function of iron-containing proteins in leaves and seeds [46]. In plants, ferritin proteins are mainly located in the plastids and barely in the cytoplasm [47]. In Arabidopsis seeds, ferritins are proposed to release Fe for the use of Fe-containing proteins after germination [46]. Beside ferritins, the vacuole is another compartment for iron storage, and NRAMP3 and NRAMP4 transport Fe from vacuole to the cytoplasm when needed. NRAMP3 and NRAMP4 are induced under Fe deficiency and functionally redundant in Arabidopsis [48,49]. The increased expression level of *NRAMP3* and decreased expression level of *FER2* and *FER3* in petunia petals under excess Cu or Fe deficiency indicate the redistribution of Fe from the storage compartment to the cytoplasm. Chloroplasts are the largest Fe storage compartment in the plant cell, containing up to 80% of total leaf Fe [2]. A NEET protein recently has been reported to participate in Fe distribution in the plant cell. The Fe content in the chloroplasts of the *neet* mutant is higher than in wild-type, and the mutant accumulates more Fe in the seedlings compared to the wild-type; however, the *neet* mutant exhibits an Fe deficiency response in its shoot. In addition, the Fe–S cluster biogenesis and chlorophyll content were also affected by the disruption of NEET function [50,51].

All these results suggest that the redistribution of Fe inside the cell is a primary response to Fe deficiency.

### 3.2. Cu Detoxification and Crosstalk with Fe in Petunia Petal under Excess Cu

Copper is translocated as Cu(II)-NA in the xylem [52]. Cu(II) needs to be reduced to Cu(I) before being taken up by shoot cells. FRO4/5 are the key proteins involved in Cu(II) reduction in Arabidopsis roots [12,33]. However, the orthologs of FRO4/5 in petunia, are barely expressed and also not induced by excess Cu in petals (Table 1), suggesting there might be another FRO protein(s) involved in Cu(II) reduction in petals, if there is any. FRO2 is the key protein involved in the Fe(III) reduction in Arabidopsis roots, which takes place at the root surface, prior to Fe uptake (as Fe(II)), and is strongly induced under Fe deficiency [33]. Petunia FRO1, an ortholog of Arabidopsis FRO1/2/3, was up-regulated in petals under excess Cu or Fe deficiency, but the fold change in Fe deficiency is much lower than that under excess Cu treatment (Figure 7). This result indicates that petunia FRO1 maybe involved in the response to excess Cu, rather than to Fe deficiency, and might primarily reduce Cu(II), rather than Fe(III).

Its reduction to Cu(I), possibly through FRO1, is expected to increase the availability of Cu for transmembrane (intracellular or cell-to-cell) transport, either as Cu^+^ or Cu(I)-NA. The cuprous ion is mainly taken up by high-affinity copper transport proteins (COPTs), and the *Petunia axillaris* genome encodes six COPT proteins, the same number as Arabidopsis, but only one, named COPT1, has been found in the DEG lists. The expression level of *COPT1* was down-regulated in petunia petals under excess Cu or Fe deficiency (Table 1 and Figure 7), indicating that it may be involved in Cu and/or Fe homeostasis. Similarly, Arabidopsis COPT2 has been reported to be involved in iron and copper crosstalk [18,53]. *AtCOPT2* encodes a plasma membrane-located Cu(I) transporter in yeast [54]. The transcripts level of *AtCOPT2* is down-regulated by excess Cu [55]. However, unlike petunia *COPT1*, *AtCOPT2* is up-regulated in the roots upon exposure to Fe deficiency [18]. In any case, the downregulation of *COPT1* in response to excess Cu in petunia petals may be taken to suggest that it is primarily a Cu transporter, possibly involved in the reloading of Cu, as Cu^+^ from the vascularity.

Beside COPTs, ZIP2 and ZIP4 are also involved in the Cu uptake in Arabidopsis [56,57], but their expression level is not effected by excess Cu in petunia petals.

The subgroup I of YSL proteins, including AtYSL1/2/3 and OsYSL16, have been reported to be involved in metal-NA transport [58]. Arabidopsis *YSL1* and *YSL3* both encode plasma membrane-located proteins and are abundantly expressed in shoots, roots and flowers. Their expression levels are down-regulated both under Fe deficiency and excess Cu [37,55]. The single mutants *ysl1* and *ysl3* have no apparent phenotypes, while the *ysl1ysl3* double mutant accumulates less Fe in the shoot when grown on MS medium [37]. Recently, it has been reported that *YSL1* and *YSL3* are the down-stream genes of the SUMO E3 ligase, SIZ1. The *siz1* mutant is more sensitive to excess Cu than wild-type. After excess Cu treatment, *siz1* mutants accumulate more Cu in the leaves, because the *siz1* mutant is unable to repress the expression of *YSL1* and *YSL3* [55]. *YSL2* encodes a plasma membrane-located protein, which is expressed in both shoots and roots, and its expression in the shoots is repressed by excess Cu and Fe deficiency [40]. YSL2 transports both Fe(II)-NA and Cu(I)-NA in yeast, but the *ysl2* mutant doesn’t show any phenotype. The Fe and Cu concentrations are not significantly different between wild type and the *ysl2* mutant, irrespective of Fe and Cu treatment levels [40]. Rice YSL16 is also involved in Cu homeostasis. *YSL16* encodes a plasma membrane-located protein, which transports Cu-NA complex in yeast. The rice *ysl16* mutant accumulates less Cu in shoots [59]. The ortholog of OsYSL16 in *Brachypodium distachyon*, BbYSL3, recently has been reported to be localized at the plasma membrane, and to mediate copper transport. The expression of *BbYSL3* was induced by Cu deficiency and repressed by Fe deficiency [60,61]. The *Petunia axillaris* genome encodes three proteins belonging to YSL1/2/3 subgroup. Petunia *YSL1* and *YSL2* were both down-regulated in Cu-stressed petals, but not affected by Fe deficiency, indicating that these two genes are involved in the homeostasis of Cu, rather than Fe.

Once inside the cell, Cu(I) interacts with Cu chaperones to avoid Cu^+^ toxicity. CCH is the only chaperone affected by excess Cu in petunia petals, in agreement with the observations in Arabidopsis shoots [62]. Heavy metal ATPase 5 (HMA5) plays an important role in Cu detoxification [8,9,10]. The *Petunia axillaris* genome encodes three HMA5 orthologs, one of HMA5I and two of HMA5II [10]. The *HMA5II* genes are barely expressed in petunia petals, and the expression of *HMA5I* is not affected by excess Cu (Table 1), which is not surprising, because its ortholog in rice, OsHMA4, is also not affected by excess Cu in shoots [8]. Another HMA superfamily protein HMA1 is involved in the delivery of Cu to the chloroplast. Under excess Cu, petunia *HMA1* was down-regulated in petals (Table 1), probably to reduce the copper uptake into the chloroplast.

Recently, it has been reported that the crosstalk between Cu and Fe in Arabidopsis root under Cu and Fe deficiency is regulated by SPL7, FIT and bHLH Ib proteins. FIT interacts with bHLH Ib proteins to activate the expression of *COPT2*, *FRO4* and *FRO5* [12,18]. Again, this is apparently not the regular network underlying the Fe and Cu crosstalk in petunia petals, because *FIT* is barely expressed in petals. There were several TFs showing significantly altered expression levels in petals under excess Cu and Fe deficiency, such as PYE2, bHLH38 and bHLH39. Although the expression level of these genes was induced by both Cu excess and Fe deficiency, the fold change in petals under excess Cu is higher than that under Fe deficiency. Besides these three TFs, one bHLH121-like protein was down-regulated in petals under excess Cu, but not affected by Fe deficiency. Arabidopsis bHLH121/URI and bHLH11 both belong to the bHLH IVb subfamily, and play important roles in the Fe deficiency response [17,63,64]. Arabidopsis *bHLH11* is mainly expressed in roots, and induced under Fe sufficient conditions. It acts as a transcriptional repressor. The expression level of *FRO2* and *FER1* was down-regulated in *bHLH11* overexpression lines, while *NRAMP4* and *NAS4* were induced in the roots of *bHLH11*-overexpressing lines under Fe deficiency [63]. In contrast to bHLH11, bHLH121 functions as a direct transcriptional activator of genes involved in the Fe deficiency response. Around 50% of the Fe homeostasis-related genes are regulated by bHLH121, including *bHLH39*, *BTS*, *BTSL1*, *BTSL2*, *YSL3*, *OPT3*, *MYB10*, *FER1*, *FER3*, *FER4*, *NEET*, *NAS4* and more [17]. Notably, the expression of these genes’ orthologs is affected by excess Cu in petunia with the same expression trends, and the expression profile in excess Cu and Fe deficiency treated petunia petals showed a strong correlation (Figure S14). The expression of *bHLH121* is not affected by the Fe status in Arabidopsis. In contrast, Fe availability affects the cellular localization of bHL121 protein in roots. However, the expression of bHLH121 orthologs are down-regulated (about 40-fold) in petunia petals under excess Cu, indicating that it might be involved in Cu homeostasis. Whether bHLH121 plays an important role in Cu and Fe crosstalk still needs to be confirmed by detailed genetic analysis.

In conclusion, excess Cu treatment induced a FIT-independent Fe deficiency response in petunia petals, with strong effects on Fe-S cluster assembling and photosynthesis. In addition, the Fe deficiency response network that controlled by bHLH121 was markedly disordered. The expression level of petinia bHLH121-like protein is significantly reduced under excess Cu, indicating it might also be involved in Cu homeostasis in petals. Further genetic studies are needed to evaluate this possibility.

## 4. Materials and Methods

### 4.1. Plant Material and Growth Conditions

Petunia seeds (cv Mitchell; W115) were sterilized with 1:10 (*v*/*v*) diluted household bleach for 10 min. Sterile seeds were stored in water in the fridge for 48 h, then seeds were sown on solid Murashige & Skoog (MS) medium. Two weeks later, the seedlings were transferred to modified half-strength Hoagland’s nutrient solution, renewed once per week [65]: 3 mM KNO_3_, 2 mM Ca(NO_3_)_2_, 1 mM K_2_HPO_4_, 0.5 mM MgSO_4_, 20 μM FeEDDHA, 50 μM KCl, 25 μM H_3_BO_3_, 2 μM ZnSO_4_, 2 μM MnSO_4_, 1 μM CuSO_4_, 0.5 μM (NH_4_)_6_Mo_7_O_24_. The pH was buffered at 5.5, using KOH and 2-(N-morpholino) ethane-sulfonic acid (MES, 2 mM). After four weeks, plants were transferred to nutrient solution supplied with 40 μM CuSO_4_ (+Cu) or without Fe (-Fe) until flowering. Then stage 4 (flower bud size 40–50 mm) and stage 7 (open flower open anthers) flowers were collected for RNA-seq and mineral analyses. Petunia was grown in a climate room (temperature around 25 °C, photon flux density at plant level 120 μmoles m^−2^s^−1^, 14 h per day, air humidity (RH) around 75%).

### 4.2. RNA-Seq

The experiments were performed with three independent biological replicates, and each replicate was a pooled sample of three individual petals. Total RNA was isolated using RNAprep pure Plant plus kit (Tiangen Biotech, China) and 1 μg total RNA from those samples was used for RNA sequencing library constructed. RNA sequencing was performed using an Illumina Novaseq 6000 instrument at GENEWIZ (Suzhou, China). In order to remove technical sequences, including adapters, polymerase chain reaction (PCR) primers, or fragments thereof, and quality of bases lower than 20, pass filter data of fastq format were processed by Cutadapt V1.9.1 (phred cutoff: 20, error rate: 0.1, adapter overlap: 1 bp, minimum length: 75, proportion of N: 0.1) to be high quality clean data. Reads were aligned to the *Petunia axillaris* genome sequence [66] using Hista2 v2.0.1. The Hisat2 parameters set were: -x Ref --dta-cufflinks -1 clean_R1.fq -2 clean_R2.fq --known-splicesite-infile splicesites.txt -p threads -S sam. Gene expression level were estimated as fragments per kilobase of transcript per million fragments (FPKM). Differential expression analysis was performed using EdgeR with adjusted *p*-value < 0.05 as a cut-off setting.

### 4.3. GO and KEGG Enrichment

The Petunia Gene Ontology (GO) annotation file was download from Plaza 4.0 [67] (http://bioinformatics.psb.ugent.be, accessed on 11 July 2021). GO and KEGG enrichment was performed using TBtools v1.098661 with *p* < 0.05 as a significance threshold [68]. Heatmaps were created using “ggplot2” package in R v4.1.0.

### 4.4. Real-Time RT-PCR

First strand cDNA was synthesized from 2 μg total RNA using PrimeScriptTM RT reagent Kit using gDNA Eraser (Perfect Real Time) (Takara). Real-time RT-PCR was performed with Green^®^ Premix Ex Taq™ II (Tli RNaseH Plus) (TaKaRa) on a Bio-Rad CFX Connect. The relative expression level was normalized to EF1α by the 2^−ΔΔCT^ method [69,70]. Primers are listed in Table S2.

### 4.5. Element Analysis

The experiments were performed with four independent biological replicates (3 individual petals per replicate). Samples were digested in H_2_O_2_/HNO_3_ (1:1, *v*/*v*) after drying at 80 °C for 48 h before measuring dry weight [71]. Minerals were analyzed using inductively coupled plasma optical emission spectrometry (ICP-OES; Optima 8000, PerkinElmer, Waltham, MA, USA).

### 4.6. Chlorophyll Content and Photochemical Activityp Measurement

Fresh petals were collected from each treatment, and chlorophyll were extracted with Acetone: Ethanol (1:1, *v*/*v*) at room temperature in the dark for 24 h. Chlorophyll content was determined photometrically by measuring absorption at 663 and 645 nm using a microplate reader (Synergy2, BioTek, Winooski, VT, USA), and was then calculated using the following formulae.

Chla (mg/g Fw) = (12.7 × A663 − 2.69 × A645) × V/1000/Fw (g)

Chlb (mg/g Fw) = (22.9 × A645 − 4.68 × A663) × V/1000/Fw (g)

Total Chl (mg/g Fw) = (20.21 × A645 + 8.02 × A663) × V/1000/Fw (g)

where V refers to the volume (mL) of extracting solution.

Dual portable fluorescence (Dual-PAM-100, WALZ, Effeltrich, Germany) was used to determine the chlorophyll fluorescence. Before measurements, all plants were kept in the dark for 30 min. A saturating pulse (20,000 μmol photons m^−2^ s^−1^, 300 ms) was applied to measure the maximum fluorescence, and then petals were illuminated at a saturating light of 358 μmol photons m^−2^ s^−1^ for 5 min to activate the electron sink in photosynthesis, and saturation pulses were generated every 20 s. PSII parameters were calculated as follows [72]: the maximum quantum yield of PSII, Fv/Fm = (Fm − Fo)/Fm; the effective quantum yield of PSII, Y(II) = (Fm’ − Fs)/Fm’; qP = F’m − F/F’m − F’o; non-photochemical quenching (NPQ) in PSII, NPQ = (Fm − Fm’)/Fm’.

### 4.7. Malondialdehyde (MDA) and Catalase (CAT) Measurement

MDA was measured using the Malondialdehyde (MDA) Content Assay Kit (Solarbio, Beijing, China), based on the thio-barbituric acid assay [73]. MDA was analyzed spectrophotometrically at 450, 532 and 600 nm. The experiments were performed with three independent biological repetitions, one petunia petal used for each measurement. CAT activity was measured using a Catalase (CAT) Activity Assay Kit (Solarbio, Beijing, China), based on the protocol of Aebi [74]. One stage 4 petal was used for each measurement, with three independent biological repetitions. The absorbance was determined at 240 nm.

### 4.8. Phylogenetic Analysis

The putative orthologs search of interesting proteins was carried out by blast search in BLAST+ v2.2.31, through TBtools v1.098661 software. Full length protein sequences were aligned using MAFFT v7.475 with default setting [75]. The alignment curation was performed using BMGE, with settings as described in Morel et al. [76]. The substitution model for the tree building was calculated with ModelFinder using the Akaike information criterion in IQ-tree v1.6.8 [77], through PhyloSuite v1.2.2 [78]. Then the phylogeny was generated using IQ-tree v1.6.8, with the parameter settings included in the descriptions of individual trees.

### 4.9. Statistical Analysis

Tukey HSD analysis was performed using the “agricolae” package in R v4.1.0. Correlation analysis between RNA-seq and qRT-PCR was also performed using R v4.1.0.

## Figures and Tables

**Table 1:** Expression profiles of genes involved in Cu and Fe homeostasis.

Gene Description	PaxiN Number	FPKM (Stage 4 CK)	FPKM (Stage 4 + Cu)	Log2 FC (Stage 4 CK versus + Cu)	FPKM (Stage 7, CK)	FPKM (Stage 7, + Cu)	Log2 FC (S7 CK versus S7 + Cu)
COPT1	Peaxi162Scf00179g00097	109.49	114.58	ND	32.25	10.52	−1.65
COPT protein	Peaxi162Scf00664g00032	0	0	ND	0.05	0	ND
COPT protein	Peaxi162Scf00765g00012	0	0	ND	0	0	ND
COPT protein	Peaxi162Scf00009g00337	0.22	0.09	ND	0	0	ND
COPT protein	Peaxi162Scf00132g00819	0.75	0.24	ND	6.54	2.57	ND
COPT5	Peaxi162Scf00486g00012	157.85	160.02	ND	369.44	351.72	ND
ZIP2	Peaxi162Scf00299g00532	0	0	ND	0.02	0	ND
ZIP4	Peaxi162Scf00158g00077	101.6	33.84	ND	93.45	56.48	ND
FRO4/5	Peaxi162Scf00444g00018	0	0	ND	0	0	ND
FRO4/5	Peaxi162Scf00444g00130	0	0	ND	0	0	ND
CCH	Peaxi162Scf00173g01010	759.64	406.96	ND	826.08	262.30	−1.71
ATX1	Peaxi162Scf01633g00011	445.43	368.77	ND	477.01	357.93	ND
CCS	Peaxi162Scf00620g00241	10.05	14.77	ND	22.80	34.69	ND
COX17-1	Peaxi162Scf00069g01430	6.68	6.39	ND	17.11	14.96	ND
COX17-2	Peaxi162Scf00027g00225	6.75	6.44	ND	9.42	6.30	ND
MT	Peaxi162Scf00362g00133	0	0	ND	6.33	13.93	ND
MT2b-1	Peaxi162Scf00774g00011	160.82	200.47	ND	1180.83	793.71	ND
MT2b-2	Peaxi162Scf00946g00028	13.05	10.60	ND	8.47	4.63	ND
HMA5I	Peaxi162Scf00029g02812	47.22	37.56	ND	31.25	30.65	ND
HMA5II-1	Peaxi162Scf00119g00510	0.11	0.12	ND	0.01	0	ND
HMA5II-2	Peaxi162Scf00119g00519	0.62	0.62	ND	0.56	0.62	ND
HMA1	Peaxi162Scf00945g00216	63.80	20.52	−1.64	30.56	18.21	ND
PAA1	Peaxi162Scf00001g01039	3.17	2.90	ND	0.82	0.67	ND
PAA2	Peaxi162Scf00486g00096	23.15	17.08	ND	19.62	17.38	ND
RAN1	Peaxi162Scf00199g00810	9.60	9.46	ND	21.06	20.17	ND
SPL7	Peaxi162Scf00095g00001	29.70	26.56	ND	28.10	23.88	ND
FIT-like	Peaxi162Scf00490g00018	0	0	ND	0	0	ND
FIT-like	Peaxi162Scf00530g00031	0	0	ND	0	0.09	ND
FIT-like	Peaxi162Scf00530g00063	0	0.06	ND	0.06	0	ND
FIT	Peaxi162Scf00530g00066	0.07	0	ND	0	0.09	ND
bHLH121-like	Peaxi162Scf00684g00019	6.89	0.75	−3.16	13.63	0.38	−5.19
bHLH11-like	Peaxi162Scf00362g01043	8.41	8.81	ND	7.58	8.57	ND
PYE1	Peaxi162Scf00274g00834	5.68	19.07	1.75	17.08	41.43	1.2
PYE2	Peaxi162Scf00712g00029	1.77	67.70	5.24	5.92	90.73	3.87
bHLH38	Peaxi162Scf00772g00076	0	4.03	9.05	0.12	64.04	9.0
bHLH39	Peaxi162Scf00772g00048	0.08	24.88	8.20	0.36	92.36	7.91
BTS1	Peaxi162Scf00753g00119	8.39	31.41	1.92	9.94	59.19	2.50
BTS2	Peaxi162Scf00021g00527	0.02	7.34	8.10	0.31	5.87	4.17
BTSL	Peaxi162Scf00009g00529	6.47	21.58	1.75	7.00	75.48	3.37
MYB10-like	Peaxi162Scf00452g00412	0.04	0.4	ND	1.06	5.95	2.44
FRO2	Peaxi162Scf00585g00115	0.00	1.35	8.63	0.02	0.37	3.74
FRO1	Peaxi162Scf00585g00014	1.14	543.08	8.88	1.68	736.18	8.71
FRO8	Peaxi162Scf00089g01433	6.75	10.38	ND	10.79	23.35	1.04
AHA2	Peaxi162Scf01054g00032	212.50	189.98	ND	249.42	205.23	ND
IRT-like	Peaxi162Scf00623g00213	0	0	ND	0	0	ND
IRT-like	Peaxi162Scf00623g00114	0	0	ND	0	0	ND
IRT-like	Peaxi162Scf00149g00128	0	0	ND	0	0.12	ND
OPT3	Peaxi162Scf00442g00710	75.43	947.23	3.65	38.56	667.36	4.03
YSL1	Peaxi162Scf00074g00232	6.29	0.27	−4.50	6.88	2.04	−1.85
YSL2	Peaxi162Scf00542g00622	15.25	3.34	−2.17	16.23	2.62	−2.72
NRAMP3	Peaxi162Scf00264g00512	68.84	293.86	2.09	151.88	460.16	1.55
NRAMP2	Peaxi162Scf00081g00618	39.30	21.30	ND	28.10	8.25	−1.83
NEET	Peaxi162Scf00192g00828	39.98	0.15	−7.94	110.74	0.27	−8.62
MFL1	Peaxi162Scf00263g00927	35.03	38.27	ND	15.67	15.52	ND
PIC1	Peaxi162Scf00016g02324	52.48	49.68	ND	44.74	47.49	ND
MIT1/2	Peaxi162Scf00390g00722	27.04	25.67	ND	26.42	28.44	ND
Ferritin3	Peaxi162Scf00284g00521	37.81	0.33	−6.79	44.99	7.04	−2.76
Ferritin2	Peaxi162Scf01149g00318	53.49	11.83	−2.16	104.08	16.55	−2.74
Ferritin1	Peaxi162Scf01132g00315	0.69	0.05	−3.55	0.34	0.00	−5.55
NAS1	Peaxi162Scf00673g00042	89.20	12.80	−2.82	38.62	7.3	−2.45
NAS2	Peaxi162Scf00750g00013	0	0	ND	0	0	ND

ND: no significant difference, fold change < 2 or FDR > 0.05.

## Data Availability

All RNA-seq raw reads have been deposited in the NCBI Sequence Read Archive (SRA) under BioProject PRJNA774370.

## Supplementary Materials

The following are available online at https://www.mdpi.com/article/10.3390/ijms222111604/s1.
