# Supplementary material for: Transcriptome Profiling of Cu Stressed Petunia Petals Reveals Candidate Genes Involved in Fe and Cu Crosstalk
Source: Int J Mol Sci. 2021 Oct 27;22(21):11604. doi: 10.3390/ijms222111604 (PMC8583722; doi:10.3390/ijms222111604)
Supplement: Supplementary file 1 [file ijms-22-11604-s001.zip › ijms-1407550-supplementary.pdf]

**Supporting Information**

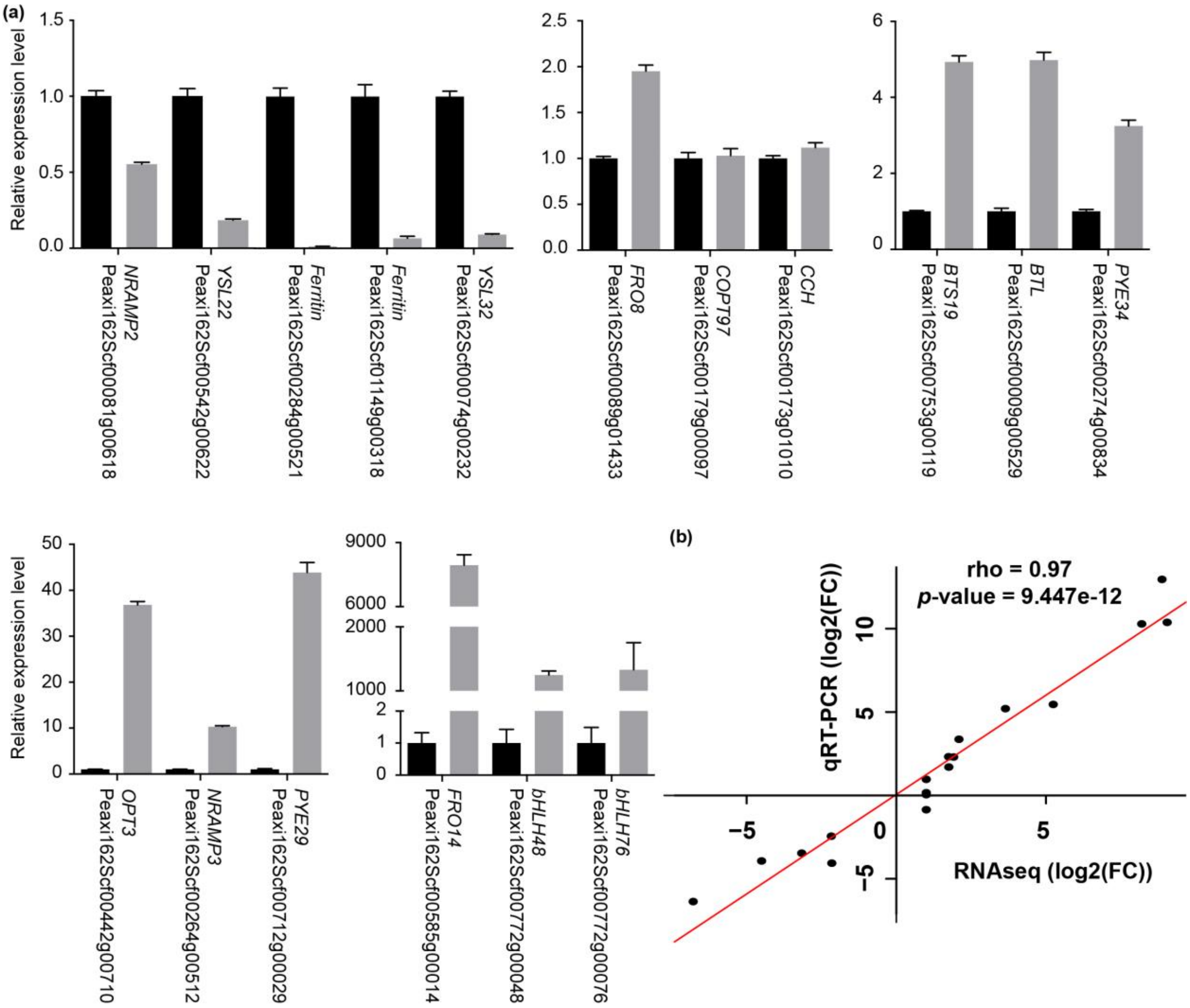


**Figure S1.** Verification of stage 4 RNA-seq data by qRT-PCR and correlation analysis. (a) The relative expression level of selected genes related to Fe homeostasis in Table 1. EF1α as the reference gene. Bars are mean ± SD (*n*=3). (b) correlation between stage 4 petal RNA-seq with qRT-PCR results. The x axis value is the log2 (fold change) from RNA-seq data. The y axis is based on the relative expression level related to untreated petal (set as 1) in (a).

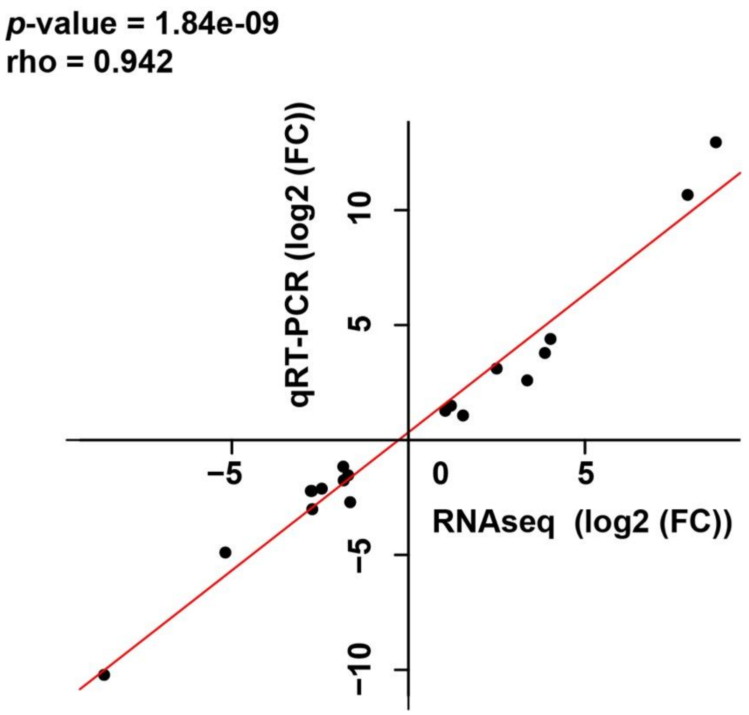


**Figure S2.** Correlation analysis result between stage 7 RNA-seq data and qRT-PCR. EF1α as the reference gene. The x axis value is the log2 (fold change) from RNA-seq data. The y axis is based on the relative expression level related to untreated petal (set as 1) in Figure 5.

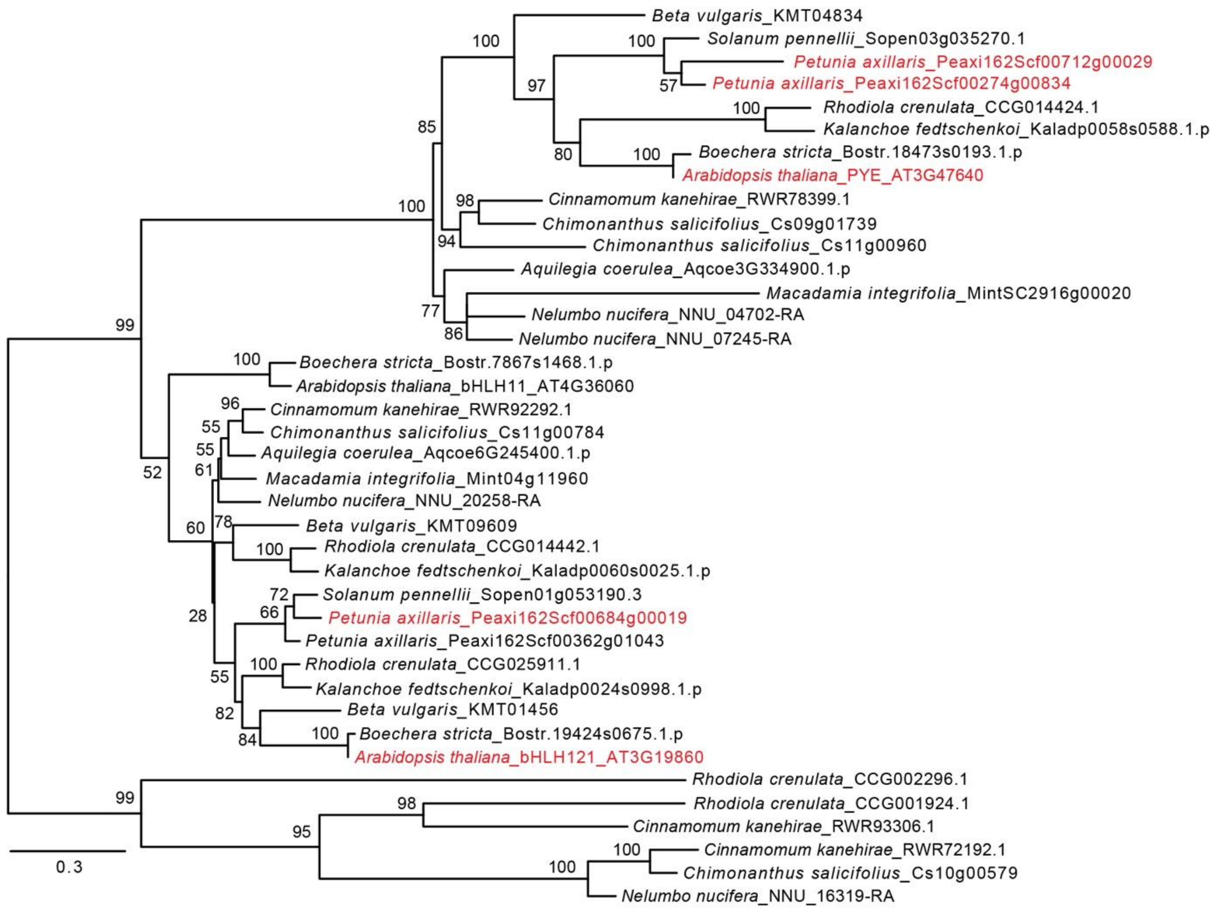


**Figure S3.** Phylogenetic tree of PYE and bHLH121 homologs from different species. Maximum likelihood phylogenies were inferred using IQ-TREE [1] under the JTT+R4 model for 1000 ultrafast bootstraps [2]. Numbers on the tree branches indicate the percentage of bootstrap support. Petunia DEGs and Arabidopsis PYE and bHLH121 are marked in red color.

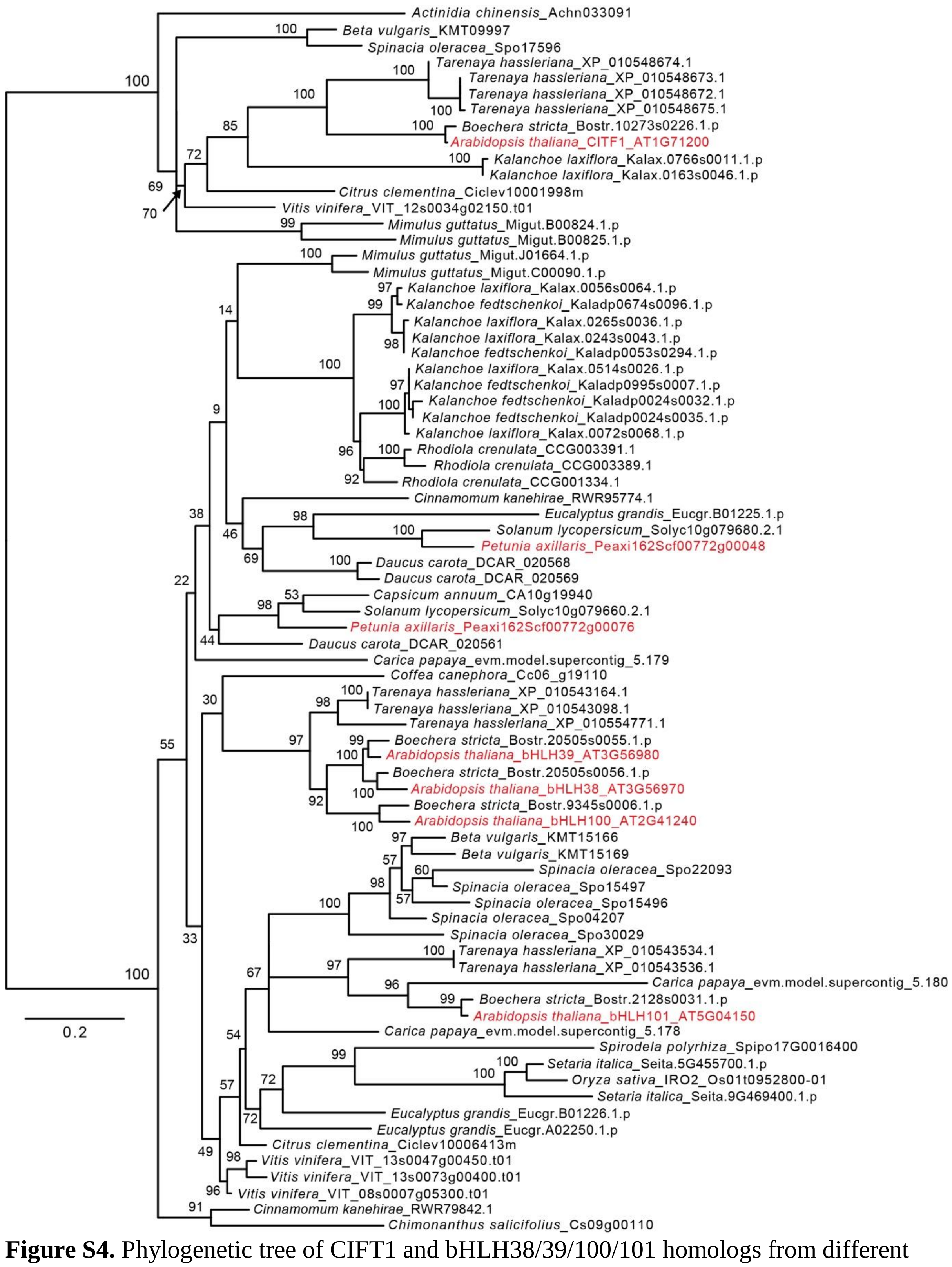


**Figure S4.** Phylogenetic tree of CIFT1 and bHLH38/39/100/101 homologs from different species. Maximum likelihood phylogenies were inferred using IQ-TREE [1] under the JTT+I+G4 model for 1000 ultrafast bootstraps [2]. Numbers on the tree branches indicate percentage of bootstrap support. Petunia DEGs, CIFT1 and bHLH38/39/100/101 are marked in red color.

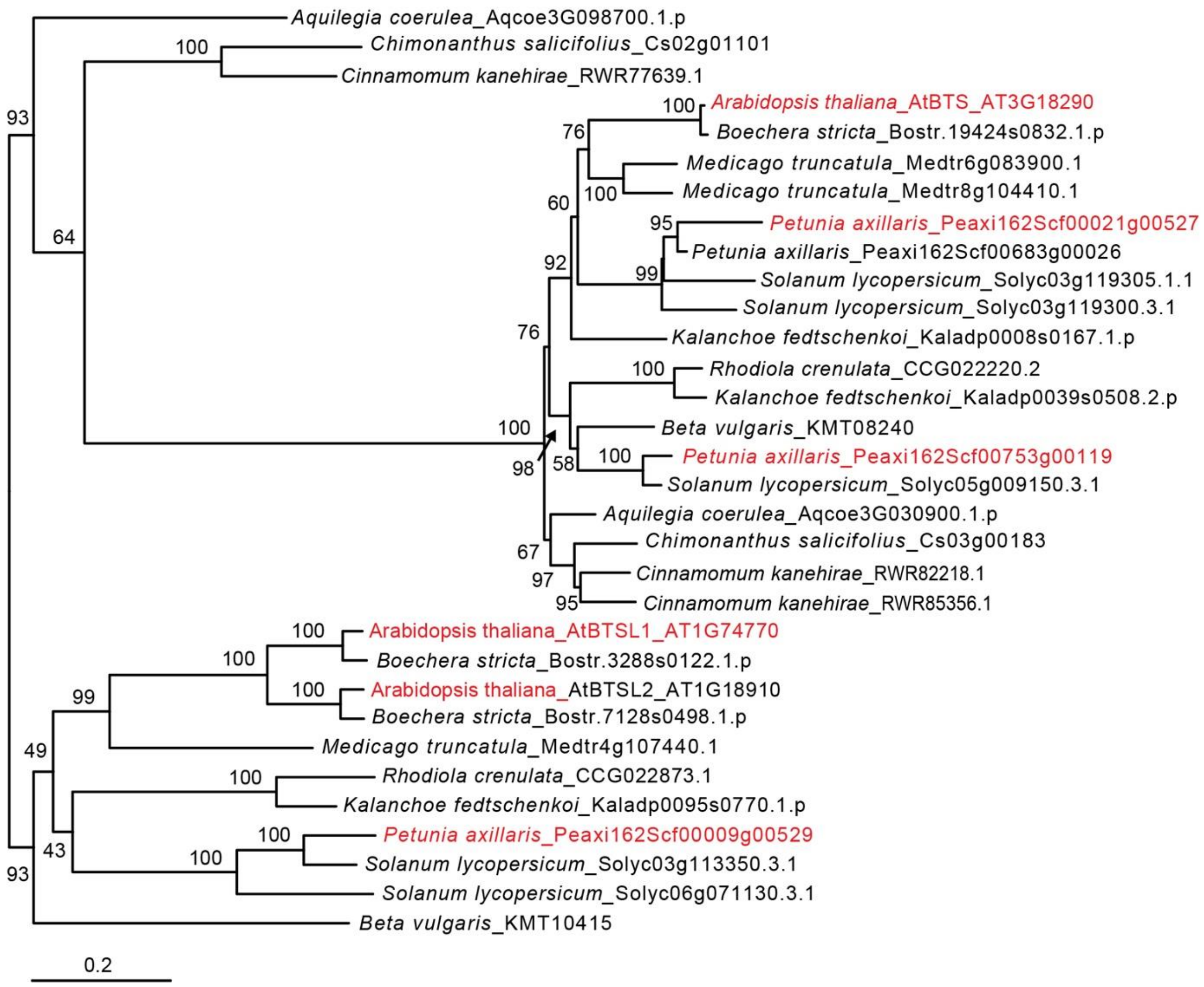


**Figure S5.** Phylogenetic tree of BTS and BTLS proteins from different species. Maximum likelihood phylogenies were inferred using IQ-TREE [1] under the JTT+R5+F model for 1000 ultrafast bootstraps [2]. Petunia DEGs and Arabidopsis BTS and BTSL are marked in red color.

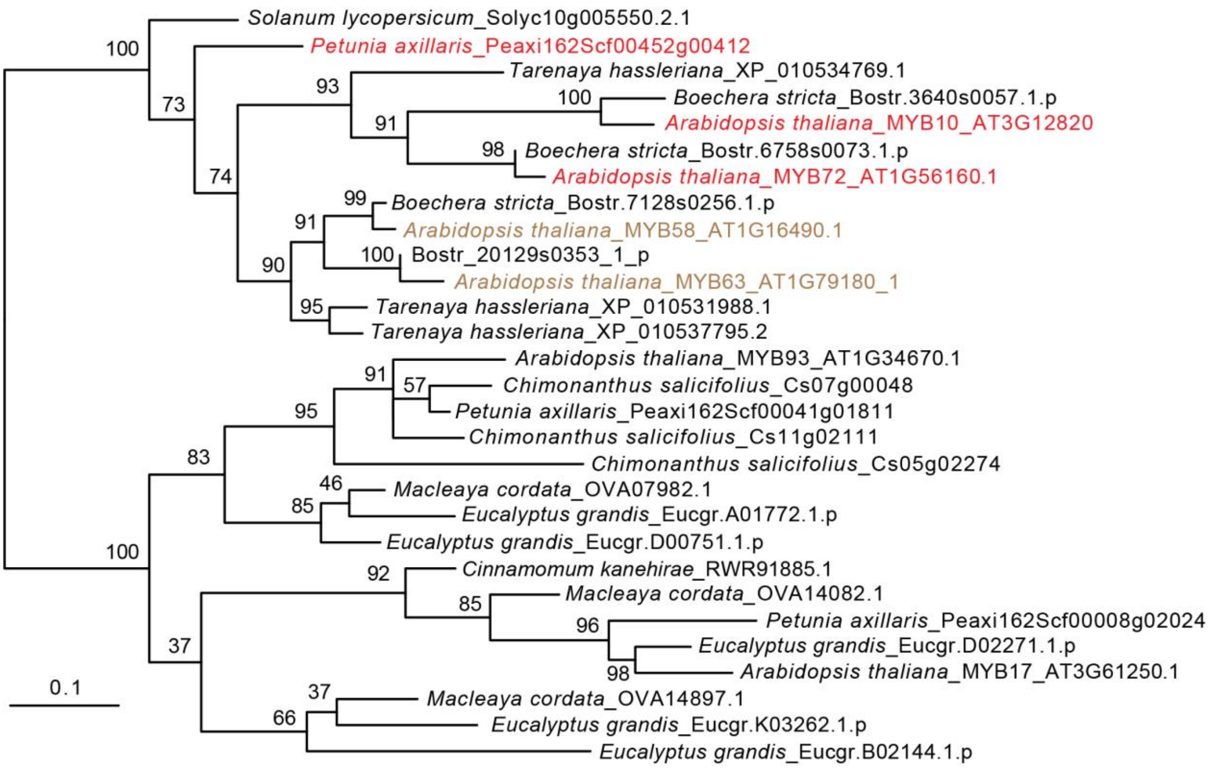


**Figure S6.** Phylogenetic tree of MYB10 orthologs. Maximum likelihood phylogenies were inferred using IQ-TREE [1] under the JTTDCMut+R4 model for 1000 ultrafast bootstraps [2], as well as the Shimodaira–Hasegawa–like approximate likelihood-ratio test [3].

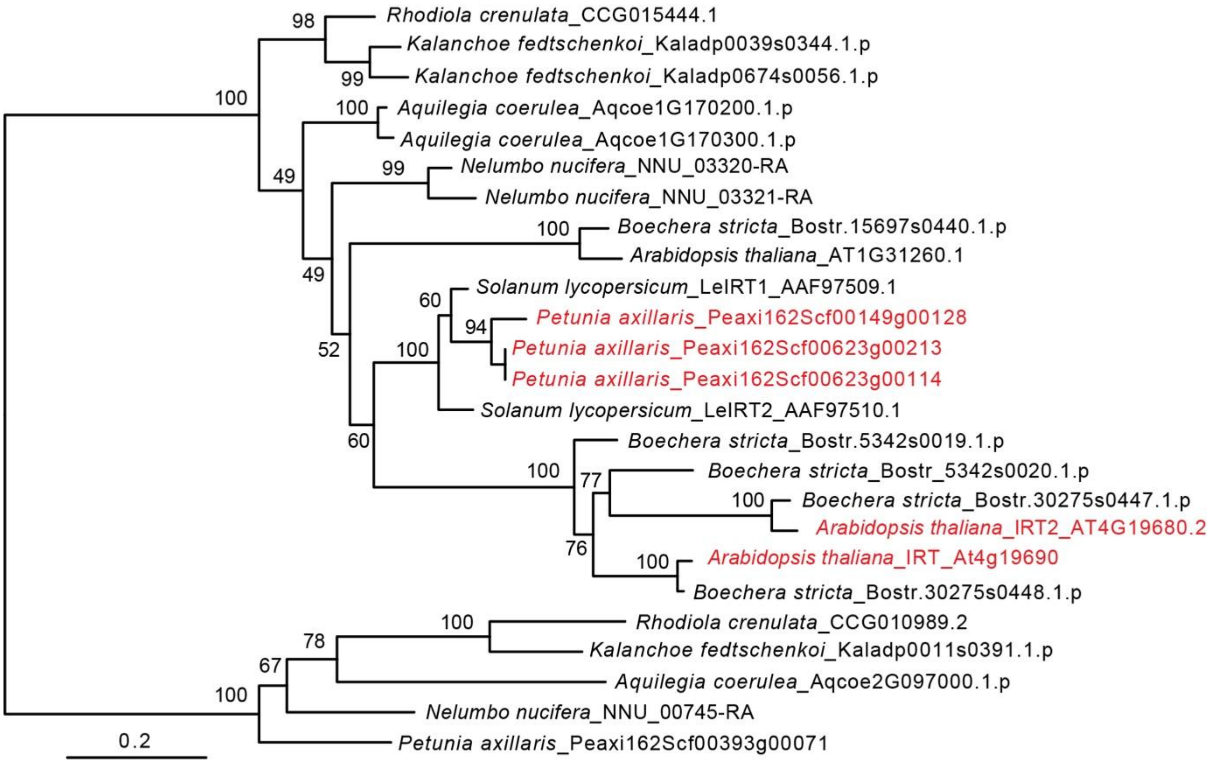


**Figure S7.** Phylogenetic tree of IRT proteins from different species. Maximum likelihood phylogenies were inferred using IQ-TREE [1] under the JTT+G4+F model for 1000 ultrafast bootstraps [2].

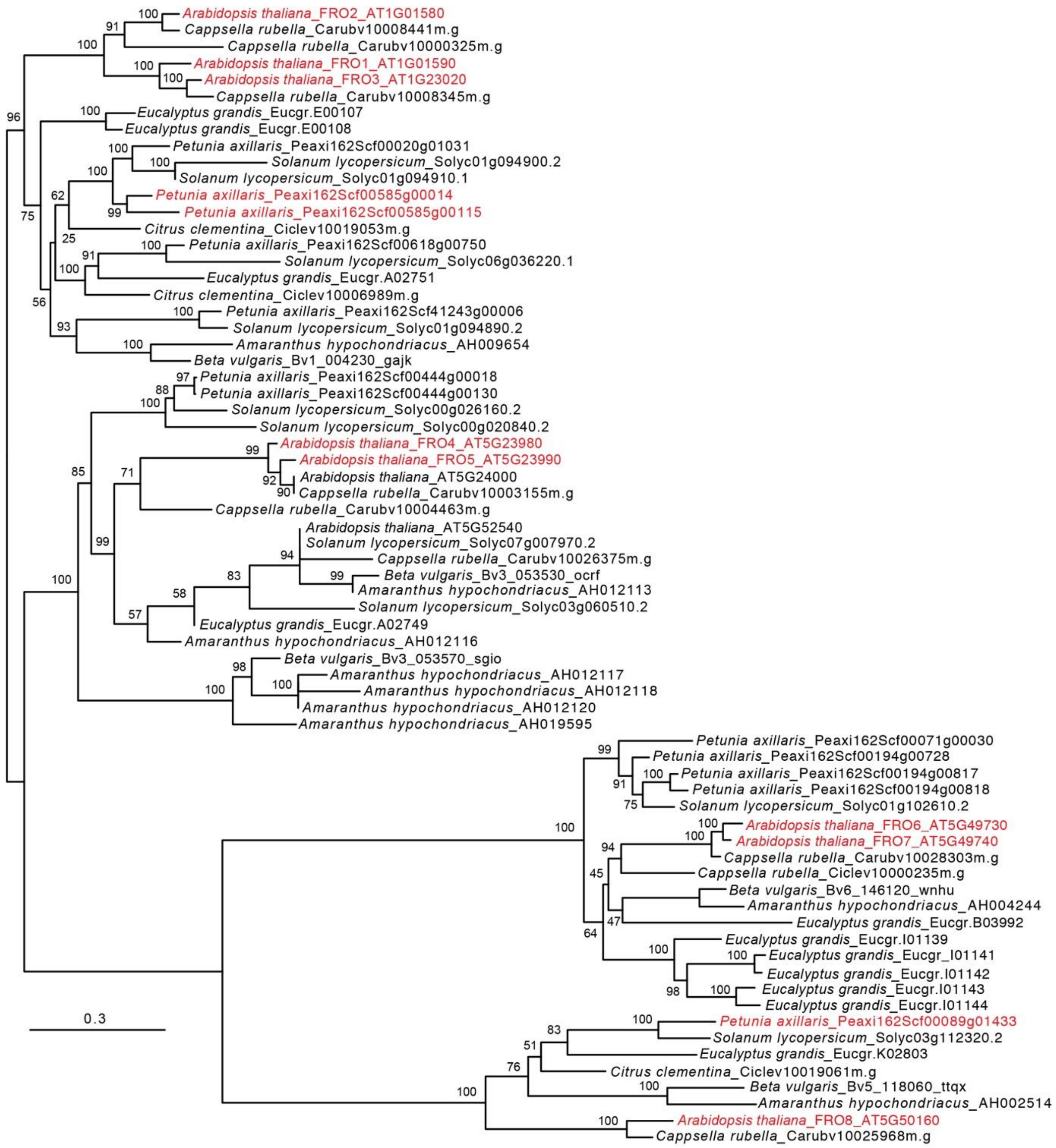


**Figure S8.** Phylogenetic tree of FRO proteins from different species. Maximum likelihood phylogenies were inferred using IQ-TREE [1] under the mtInv+R5+F model for 1000 ultrafast bootstraps [2].

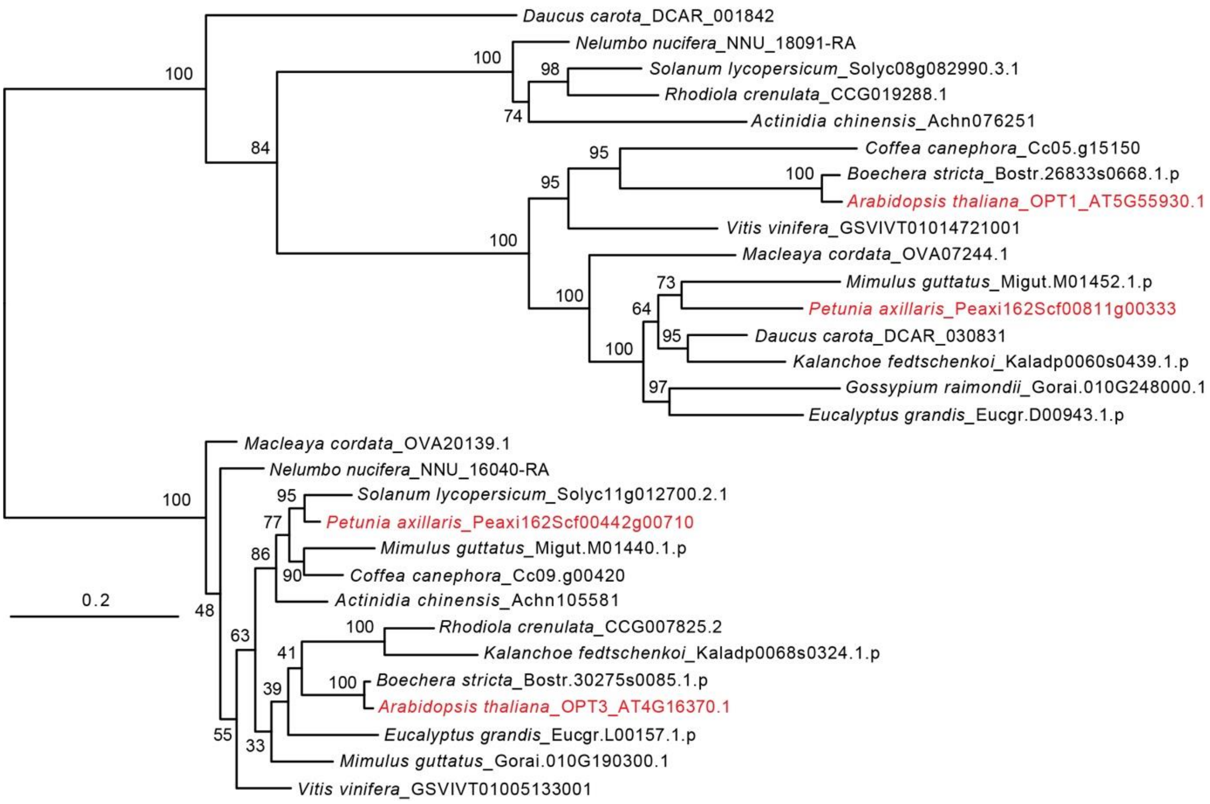


**Figure S9.** Phylogenetic tree of OPT3 proteins from different species. Maximum likelihood phylogenies were inferred using IQ-TREE [1] under the LG+R4 model for 1000 ultrafast bootstraps [2].

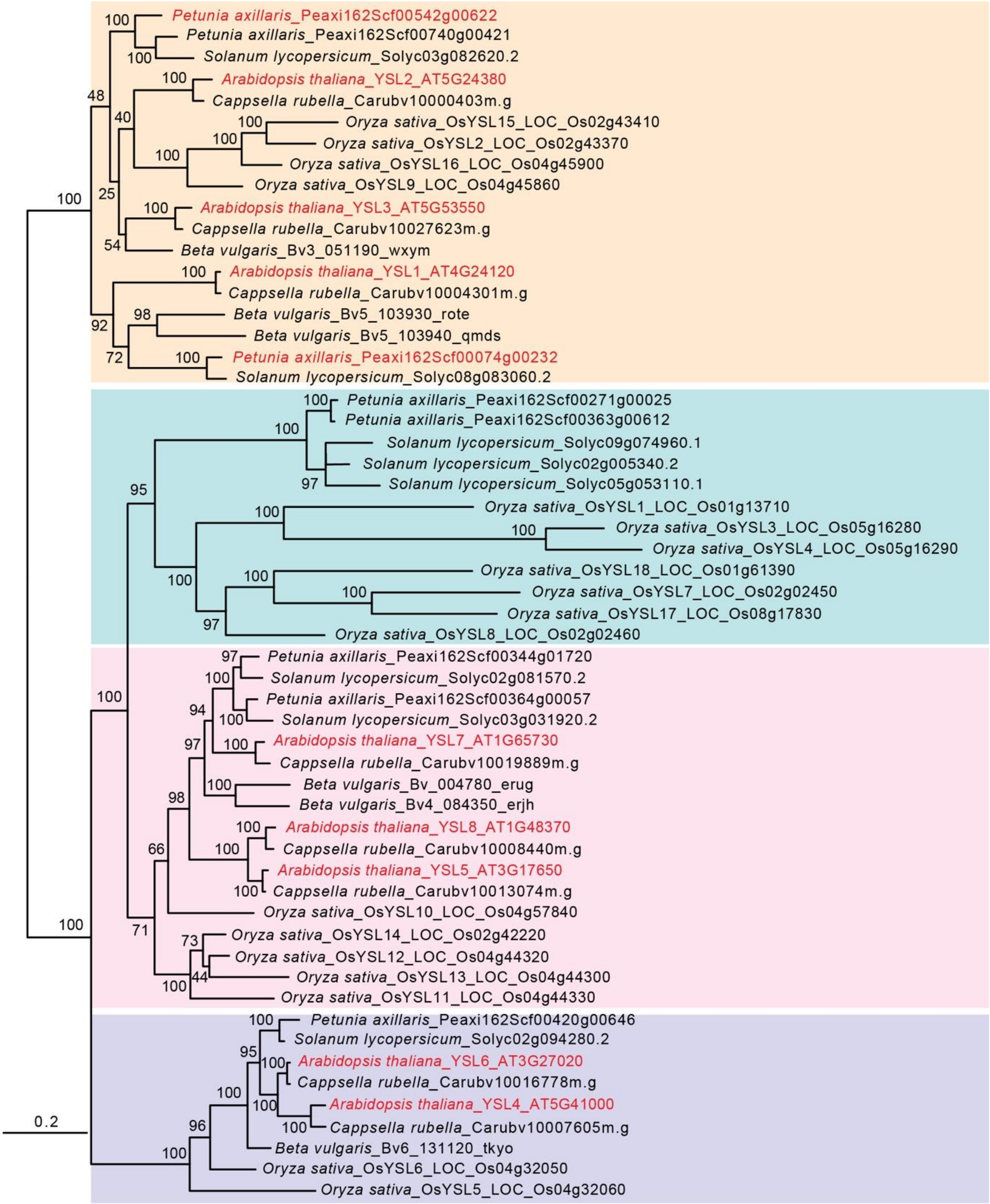


**Figure S10.** Phylogenetic tree of a selection of YSL proteins. Maximum likelihood phylogenies were inferred using IQ-TREE [1] under the LG+R5+F model for 1000 ultrafast bootstraps [2].

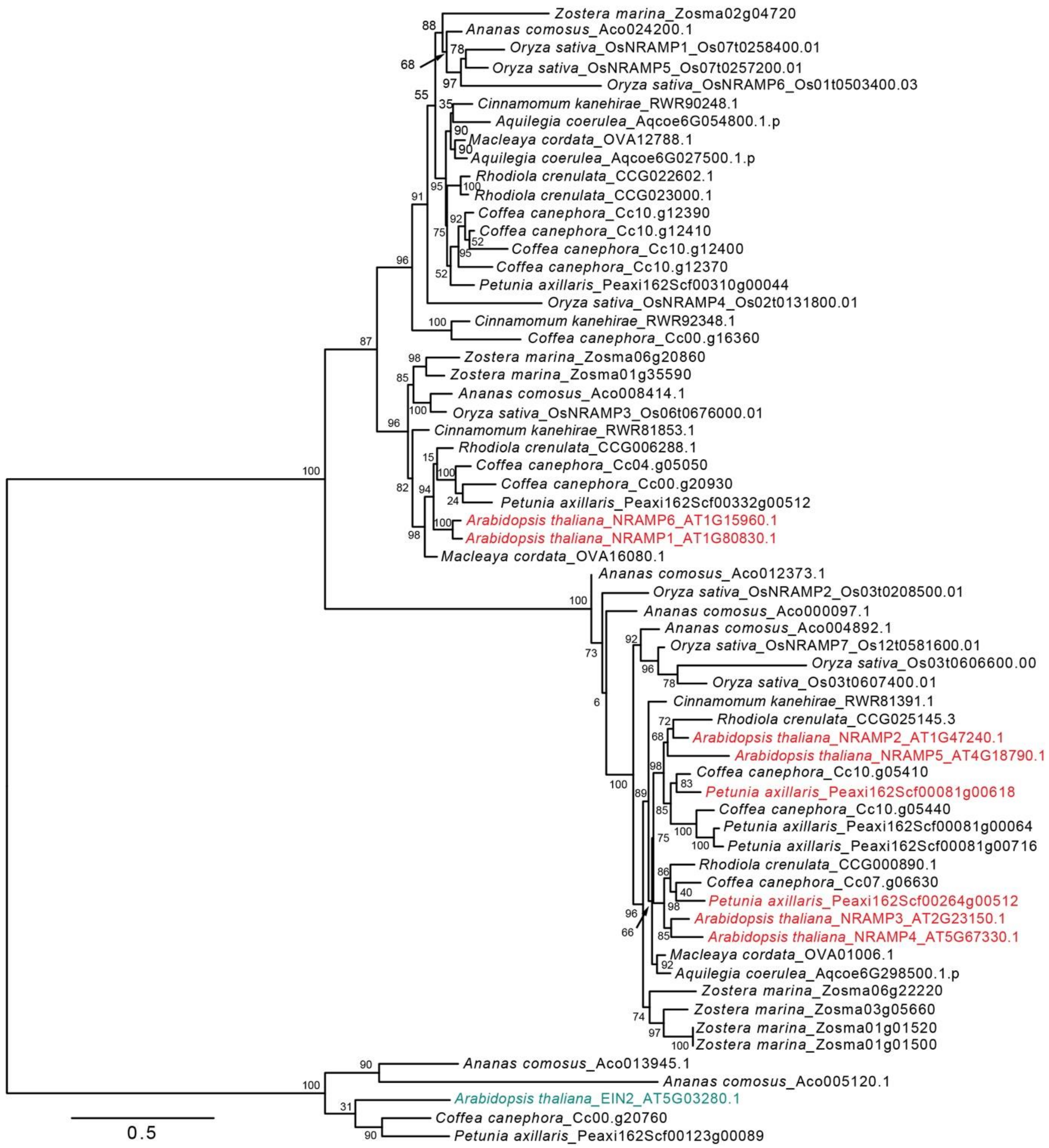


**Figure S11.** Phylogenetic tree of NRAMP proteins from different species. Maximum likelihood phylogenies were inferred using IQ-TREE [1] under the JTT+R4+F model for 1000 ultrafast bootstraps [2].

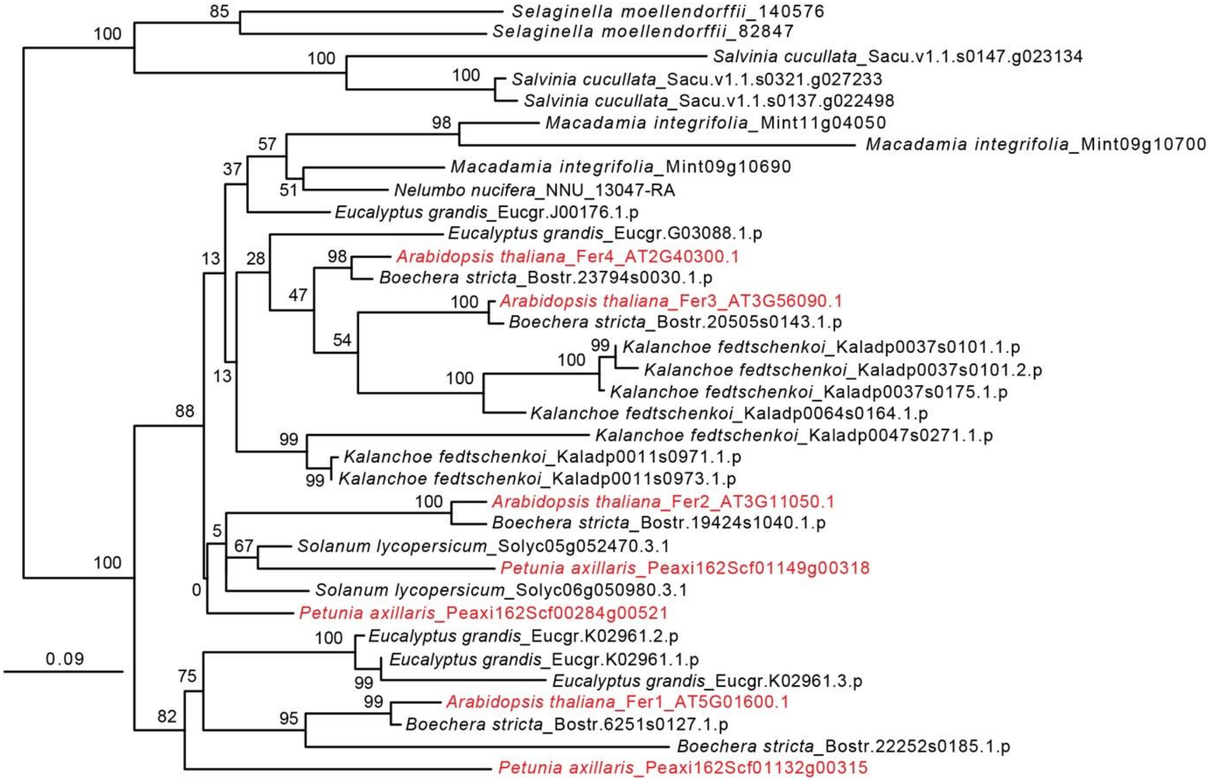


**Figure S12.** Phylogenetic tree of Ferritin proteins from different species. Maximum likelihood phylogenies were inferred using IQ-TREE [1] under the JTT+R4 model for 1000 ultrafast bootstraps [2].

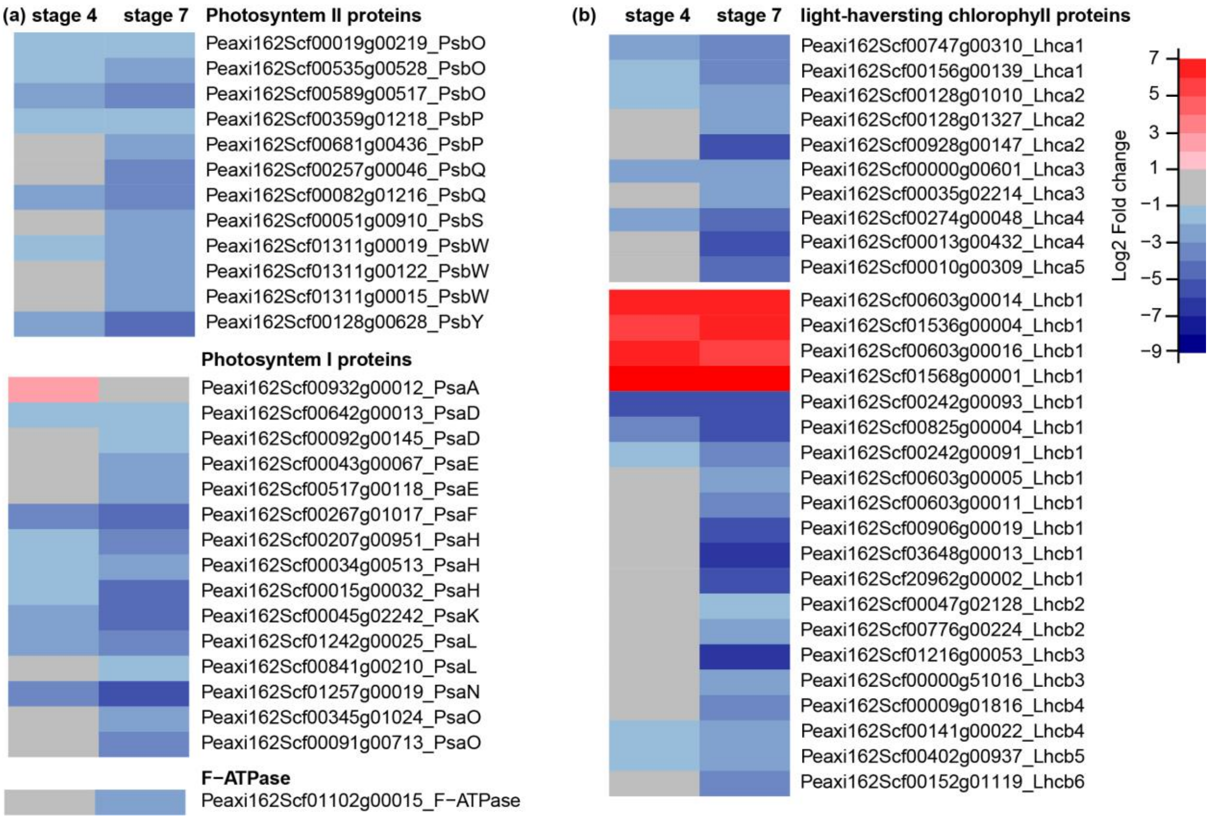

**Figure S13**. Heatmap showing the expression profile of genes involved in photosynthesis. (a) and photosynthesis-antenna proteins (b), according to the KEGG enrichment analysis.

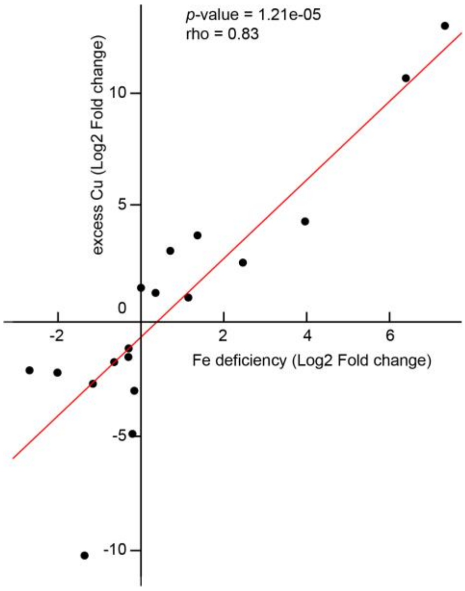


**Figure S14.** Correlation analysis between qRT-PCR results of petunia petals under excess Cu and Fe deficiency. Log 2 Fold change of gene expression was calculated using the relative expression level of qRT-PCR data in Figure 7.

**Table S1.** Expression profiling of genes involved in chlorophyll biosynthesis.

| | | FPKM | | | |
|---|---|---|---|---|---|
| | | stage 4 | | stage 7 | |
| **Gene** | **PaxiN ID** | **control** | **+Cu** | **control** | **+Cu** |
| GluRS | Peaxi162Scf00362g00137 | 31.21 | 30.57 | 48.78 | 46.44 |
| HEMA1 | Peaxi162Scf00299g00234 | 0.70 | 0.48 | 1.27 | 0.08 |
| HEMA2 | Peaxi162Scf00626g00719 | 19.21 | 6.09 | 47.92 | 12.16 |
| GSA1 | Peaxi162Scf00681g00117 | 28.62 | 25.05 | 19.34 | 18.15 |
| GSA2 | Peaxi162Scf00334g00121 | 7.58 | 6.11 | 5.74 | 4.27 |
| ALAD | Peaxi162Scf00288g00822 | 46.29 | 45.06 | 31.06 | 29.56 |
| PBGD-1 | Peaxi162Scf00452g00831 | 20.73 | 20.81 | 6.76 | 9.49 |
| PBGD-2 | Peaxi162Scf00006g00139 | 19.64 | 20.51 | 36.74 | 37.23 |
| UROS1 | Peaxi162Scf00566g00219 | 10.60 | 8.48 | 15.90 | 11.10 |
| UROS2 | Peaxi162Scf00304g00417 | 31.21 | 28.75 | 41.81 | 32.50 |
| UROS3 | Peaxi162Scf00252g00416 | 9.44 | 9.89 | 4.54 | 4.30 |
| UROD | Peaxi162Scf00692g00610 | 7.77 | 6.01 | 7.74 | 4.90 |
| CPOX | Peaxi162Scf00005g00134 | 4.05 | 3.24 | 1.97 | 1.36 |
| PPOX1 | Peaxi162Scf00309g00623 | 12.05 | 12.31 | 18.92 | 18.90 |
| PPOX2 | Peaxi162Scf00700g00151 | 24.07 | 22.98 | 55.74 | 47.05 |
| MgCh1 | Peaxi162Scf00130g00022 | 5.52 | 2.42 | 12.51 | 1.95 |
| MgCh2 | Peaxi162Scf01713g00016 | 18.66 | 16.37 | 9.69 | 7.17 |
| MgCh3 | Peaxi162Scf00269g00514 | 36.50 | 24.44 | 30.96 | 10.86 |
| MgMT | Peaxi162Scf00314g00625 | 2.69 | 1.23 | 9.66 | 2.91 |
| MPEC | Peaxi162Scf00003g05355 | 13.23 | 10.34 | 105.75 | 57.58 |
| POR A | Peaxi162Scf00000g43015 | 0.00 | 0.00 | 0.03 | 0.01 |
| POR B | Peaxi162Scf00132g00524 | 6.63 | 4.39 | 6.20 | 1.10 |
| POR C | Peaxi162Scf00027g00303 | 3.56 | 2.37 | 7.32 | 4.30 |
| DVR1 | Peaxi162Scf00386g00096 | 2.71 | 4.12 | 7.44 | 4.98 |
| CAO | Peaxi162Scf00051g01011 | 7.06 | 54.38 | 18.94 | 87.63 |
| CHLG | Peaxi162Scf00890g00119 | 15.29 | 12.47 | 22.85 | 17.09 |

**Table S2.** Primers sequences used for qRT-PCR

| Gene | Primer ID | Sequence 5'-3' | Orientation |
|---|---|---|---|
| *EF1α* | 062R | ATCCTGGTCAAATTGGAAACGG | forward |
| *EF1α* | 063R | CAGATCGCCTGTCAATCTTGG | reverse |
| *BTSL* | 0644 | CTCTATCTGATCACACATGCAG | forward |
| *BTSL* | 0645 | CAGATGACCACAAGGAAGTGC | reverse |
| *NAS1* | 0612 | GCGTTGGTTGGTATGGATATC | forward |
| *NAS1* | 0613 | GCACCATGTGCACTCCTTAG | reverse |
| *OPT3* | 0616 | TTGAGACTCTTCATCCAGACAG | forward |
| *OPT3* | 0617 | AACCGCTCTGGTCCAATCAG | reverse |
| *FER3* | 0622 | CTTGTACCTACTGTTCCACAAG | forward |
| *FER3* | 0623 | AAACATCGCGTGATAAACATACG | reverse |
| *FER2* | 0664 | GAGGACATGCTGAGAAATTGATG | forward |
| *FER2* | 0665 | ACAAACTTACCATACAACGCATC | reverse |
| *NRAMP3* | 0628 | CAGGTCAATATCTTCAGGATAAG | forward |
| *NRAMP3* | 0629 | CAGCATAAGTGCCAGTTATAGTG | reverse |
| *bHLH121-like* | 0656 | GCCTGAATGAACAGTTCACTG | forward |
| *bHLH121-like* | 0657 | CAGCACTTGAATGGTATCACTG | reverse |
| *PYE1* | 0648 | AATTGTTCACAAGGCTGAGAGAG | forward |
| *PYE1* | 0649 | CCATTCGTTTGCTCTGATAATTC | reverse |
| *PYE2* | 0652 | CTATCTGAGCAGAATCAGCAG | forward |
| *PYE2* | 0653 | TCCTCAGATGCTTGATCTGAG | reverse |
| *BTS1* | 0640 | TCGCTGGATAGCTGCTCAG | forward |
| *BTS1* | 0641 | AACTTGCTTGTCTGGATCACG | reverse |
| *YSL1* | 0632 | GTTCCTCTCCCAATGGCTATG | forward |
| *YSL1* | 0633 | ATCAAACTAGCCTTCCTGCTG | reverse |
| *YSL2* | 0636 | TTGCAGCTCTGTTATGGCTTC | forward |
| *YSL2* | 0637 | CACAGCCATTGCCATAGGCAG | reverse |
| *NRAMP2* | 0626 | GTGCTTCAGTCTATACAGATC | forward |
| *NRAMP2* | 0627 | GCAACAGTCCATGCAACTCTC | reverse |
| *FRO8* | 0660 | CATCGATGGAGGTCCGTAAG | forward |
| *FRO8* | 0661 | GTTATGCAGTATATGGAGTTGAG | reverse |
| *bHLH038* | 0574 | AATAATTCATCTCCTCCATCTCAC | forward |
| *bHLH038* | 0575 | CTGCGCTCACTTGCGTTATG | reverse |
| *bHLH039* | 0534 | AATGTTCTGTCTTGGAGATTGTTC | forward |
| *bHLH039* | 0682 | TCTCAAGTACATACCCGAGTTAC | reverse |
| *FRO1* | 0525 | GCCAATTACTCCAATTCTTGGA | forward |
| *FRO1* | 0526 | CGATTGGGTAGATGTAATAACTAG | reverse |
| *NEET* | 0602 | AGCTAAAGTTGTGGACTCTGTTG | forward |
| *NEET* | 0603 | TCACATGGCTTCCATCACATAG | reverse |
| *COPT1* | 0668 | CGCCATGAACAACATGACAATG | forward |
| *COPT1* | 0669 | CATAACCAGGCCAACCTGAG | reverse |
| *CCH* | 0672 | GTCTCAGACAGTTGTTCTCAAG | forward |
| *CCH* | 0673 | TCCACATTTCCCTTAACAGTCAC | reverse |

**Reference:**


1. Nguyen, L.-T.; Schmidt, H.A.; von Haeseler, A.; Minh, B.Q. IQ-TREE: a fast and effective stochastic algorithm for estimating maximum-likelihood phylogenies. *Mol. Biol. Evol.* **2015**, *32*, 268–274, doi:10.1093/molbev/msu300.
2. Minh, B.Q.; Nguyen, M.A.T.; von Haeseler, A. Ultrafast approximation for phylogenetic bootstrap. *Mol. Biol. Evol.* **2013**, *30*, 1188–1195, doi:10.1093/molbev/mst024.
3. Guindon, S.; Dufayard, J.F.; Lefort, V.; Anisimova, M.; Hordijk, W.; Gascuel, O. New algorithms and methods to estimate maximum-likelihood phylogenies: Assessing the performance of PhyML 3.0. *Syst. Biol.* **2010**, *59*, 307–321, doi:10.1093/sysbio/syq010.
